# Combination of oral STING agonist MSA-2 and anti-TGF-β/PD-L1 bispecific antibody YM101: a novel immune cocktail therapy for non-inflamed tumors

**DOI:** 10.1186/s13045-022-01363-8

**Published:** 2022-10-08

**Authors:** Ming Yi, Mengke Niu, Yuze Wu, Hong Ge, Dechao Jiao, Shuangli Zhu, Jing Zhang, Yongxiang Yan, Pengfei Zhou, Qian Chu, Kongming Wu

**Affiliations:** 1 Department of Oncology, Tongji Hospital of Tongji Medical College, Huazhong University of Science and Technology, 1095 Jiefang Avenue, Wuhan, 430030 People’s Republic of China; 2 Department of Breast Surgery, The First Affiliated Hospital, College of Medicine, Zhejiang University, Hangzhou, 310000 People’s Republic of China; 3 Department of Radiation Oncology, The Affiliated Cancer Hospital of Zhengzhou University & Henan Cancer Hospital, Zhengzhou, 450008 People’s Republic of China; 4 Department of Interventional Radiology, The First Affiliated Hospital of Zhengzhou University, Zhengzhou, 450052 People’s Republic of China; 5 Wuhan YZY Biopharma Co., Ltd, C2-1, No.666 Gaoxin Road, Biolake, Wuhan, 430075 People’s Republic of China

**Keywords:** STING, PD-1, PD-L1, TGF-β, Cancer immunotherapy, Bispecific antibody, The tumor microenvironment

## Abstract

**Background:**

Non-inflamed tumors, including immune-excluded and immune-desert tumors, are commonly resistant to anti-PD-1/PD-L1 (α-PD-1/PD-L1) therapy. Our previous study reported the potent antitumor activity of anti-TGF-β/PD-L1 bispecific antibody YM101 in immune-excluded tumors. However, YM101 had limited antitumor activity in immune-desert models. MSA-2 is a novel oral stimulator of interferon genes (STING) agonist, which activates the innate immune system and may synergize with YM101 in overcoming immunotherapy resistance.

**Methods:**

The dose-dependent effect of MSA-2 on STING signaling was determined by interferon-β level. The maturation and function of dendritic cell (DC) were measured by flow cytometry, RNA-seq, one-way mixed lymphocyte reaction (MLR), OVA peptide pulse, and cytokine/chemokine detection. The synergistic effect between MSA-2 and YM101 was assessed by one-way MLR. The macrophage activation was measured by flow cytometry and cytokine/chemokine detection. The in vivo antitumor activity of MSA-2 combined with YM101 was explored in syngeneic murine tumor models. After treatments, the alterations in the tumor microenvironment (TME) were detected by flow cytometry, immunohistochemistry staining, immunofluorescence staining, RNA-seq, and single-cell RNA-seq (scRNA-seq).

**Results:**

MSA-2 could promote the maturation and antigen presentation capability of murine DC. In the one-way MLR assay, MSA-2 synergized with YM101 in enhancing naive T cell activation. Moreover, MSA-2 stimulated the classical activation of macrophage, without significant influence on alternative activation. Further in vivo explorations showed that MSA-2 increased multiple proinflammatory cytokines and chemokines in the TME. MSA-2 combined with YM101 remarkedly retarded tumor growth in immune-excluded and immune-desert models, with superior antitumor activity to monotherapies. Flow cytometry, bulk RNA-seq, and scRNA-seq assays indicated that the combination therapy simultaneously boosted the innate and adaptive immunity, promoted antigen presentation, improved T cell migration and chemotaxis, and upregulated the numbers and activities of tumor-infiltrating lymphocytes.

**Conclusion:**

Our results demonstrate that MSA-2 synergizes with YM101 in boosting antitumor immunity. This immune cocktail therapy effectively overcomes immunotherapy resistance in immune-excluded and immune-desert models.

**Supplementary Information:**

The online version contains supplementary material available at 10.1186/s13045-022-01363-8.

## Background

The cancer-immunity cycle consists of cascading events starting with cancer antigen release and ending with cancer killing. In patients with cancer, this cycle is halted by several negative factors such as programmed cell death protein 1 (PD-1) [1]. The engagement of PD-1 with its ligands PD-L1/2 counteracts T cell activation triggered by T cell receptor (TCR) signaling [2]. Blocking PD-1/PD-L1 reinvigorates exhausted T cells and promotes immune normalization in the tumor microenvironment (TME) [3]. The FDA has approved PD-1/PD-L1 blockade therapy for more than ten cancer indications [4]. However, most cancer patients respond poorly to α-PD-1/PD-L1 monotherapy [5, 6]. For these non-responders, other negative factors, such as transforming growth factor-β (TGF-β), dominate the balance between immunity and tolerance, so it is insufficient to reprogram antitumor immunity by α-PD-1/PD-L1 [7–9].

According to the status of tumor-infiltrating T cells, tumors are commonly classified into three subtypes: immune-inflamed (T cells are in close proximity to tumor cells), immune-excluded (T cells are embedded in tumor stroma), and immune-desert tumors (devoid of T cell infiltration) [10]. Among three subtypes, immune-inflamed tumors are more likely to respond to α-PD-1/PD-L1 therapy. Conversely, immune-inflamed and immune-desert tumors are frequently resistant to α-PD-1/PD-L1 therapy. Immune-inflamed tumors are associated primarily with hyperactive TGF-β signaling, increased stromal generation, dysregulated tumor angiogenesis, and myeloid-derived suppressor cells. Immune-desert tumors show active tumor cell proliferation, enhanced fatty acid metabolism, and neuroendocrine features [11]. Notably, due to intratumoral immune heterogeneity, immune-inflamed, immune-excluded, or immune-desert entities could potentially coexist in different areas of the same tumor [12–15].

TGF-β is a versatile cytokine regulating multiple components in the cancer-immunity cycle: undermining T cell proliferation and activation, hampering the activities of DC and NK cell, promoting regulatory T (Treg) cell differentiation, and enhancing cancer-associated fibroblast (CAF) activities [16–18]. Hyperactive TGF-β signaling in the TME is associated with the resistance to α-PD-1/PD-L1, and the combination of α-TGF-β with α-PD-1/PD-L1 effectively overcomes treatment resistance in immune-excluded models [19–21]. Based on the synergistic effect of α-TGF-β and α-PD-L1, we developed a bispecific antibody (BsAb) YM101 targeting TGF-β and PD-L1 [22]. YM101 had potent antitumor activity in immune-inflamed and immune-excluded models, but its efficacy was limited in immune-desert models [23].

Immune-desert tumors are characterized as deficient antigen presentation and lack of immune infiltration [10]. Strengthening immunogenic cancer cell death or antigen presentation cell (APC) function could relieve the low immunogenicity-mediated α-PD-1/PD-L1 resistance [24]. Stimulator of interferon genes (STING) agonists promote DC maturation and synergize with α-PD-1/PD-L1 in mice and patients [25–28]. However, the delivery of conventional STING agonists depends on intratumoral injection, limiting their application in the clinic [28, 29]. MSA-2 is an oral non-nucleotide STING agonist, resolving the delivery flaw of conventional STING agonists [30]. MSA-2 is a milestone in cancer immunotherapy, providing a promising adjuvant suitable for systemic administration. Considering the critical role of STING pathway in triggering the cancer-immunity cycle, we presumed that MSA-2 would enhance YM101 efficacy in immune-excluded tumors and alleviate YM101 resistance in immune-desert tumors. In this study, we evaluated the antitumor activity of MSA-2 combined with YM101 in multiple types of non-inflamed tumors. Besides, we analyzed the immune profile of the TME after monotherapy or combination therapy, aiming to reveal the atlas of tumor-infiltrating immune cell repertoire and the mechanisms of synergistic effects.

## Materials and methods

### Cell lines and therapeutic Abs

Murine cancer cell lines B16, CT26, EMT-6, and H22, were cultured in RPMI-1640 (Gibco) with 10% fetal bovine serum (Gibco). Abs used in this study included human IgG (hIgG, isotype control), IGY targeting PD-L1, Y100-C4 targeting TGF-β, and YM101 targeting PD-L1 and TGF-β BsAb. All Abs were obtained from Wuhan YZY Biopharma.

### Induction of murine bone marrow-derived DC (BMDC) and bone marrow-derived macrophage (BMDM)

BMDC was induced as previously described. Briefly, bone marrow cells were harvested from BALB/c mice and cultured with the differentiation medium. The differentiation medium contained 20 ng/ml murine granulocyte–macrophage colony-stimulating factor (GM-CSF) (315-03-50UG, PeproTech), 20 ng/ml murine IL-4 (214-14-50UG, PeproTech), and penicillin–streptomycin (1:100, 15,140-22, Gibco). On day 2, the suspension cells were discarded, and the fresh differentiation medium was added. On day 5, the suspension cells were retained after centrifugation, and 200 ng/ml lipopolysaccharides (LPS) (14011S, CST) or MSA-2 (HY-136927, MCE) was added. On day 6, cells and supernatant were collected. DC maturation was evaluated by CD80, CD86, H-2Kd, and HLA-DR levels.

BMDM was induced from bone marrow cells using 20 ng/ml murine macrophage colony-stimulating factor (M-CSF) (315-02-100UG, PeproTech). On day 2, the suspension cells were discarded, and the fresh differentiation medium was added. On day 5, the supernatant was replaced, and 200 ng/ml LPS (positive control for classical activation), 20 ng/ml murine IL-4 (positive control for alternative activation), or MSA-2 were added [25]. On day 6, cells and supernatant were collected. Macrophage activation was assessed by flow cytometry. Abs targeting CD11c (553,801, BD), CD80 (560,016, BD), CD86 (105,008, BioLegend), H-2Kd (742,436, BD), HLA-DR (307,606, BioLegend), PD-L1 (124,312, BioLegend), F4/80 (123,120, BioLegend), and CD206 (141,708, BioLegend) were used in the assays. Cell viability was measured by AO/PI double staining (CS2-0106-5ML, Nexcelom).

### In vitro cytokine and chemokine detection

The supernatants of BMDC and BMDM were harvested for cytokine and chemokine detection. Murine IFN-β level was measured by enzyme-linked immunosorbent assay (ELISA) (MIFNB0, R&D). Other murine cytokines and chemokines were detected by multiplex fluorescence-encoded beads (741,044, BioLegend; 740,451, BioLegend).

### One-way mixed lymphocyte reaction (MLR)

Stimulating cells were BMDCs derived from BALB/c mice while responding cells were spleen cells from C57BL/6 in the MLR assays. The stimulating cells were activated with 200 ng/ml LPS or 0.01 mg/ml MSA-2 for one day, and then pretreated with 50 μg/ml Mitomycin-C (S8146, Selleck) for 20 min. The responding cells were prestained with 5 μM carboxyfluorescein diacetate succinimidyl ester (CFSE) (65–0850-84, ThermoFisher). The mixed cells (the ratio of stimulator to responder = 1:2) were cultured for four days. On day 5, the supernatants and mixed cells were collected for CFSE dilution assay and cytokine detection. Abs targeting CD4 (100,516, BioLegend) and CD8 (563,068, BD) were used in the flow cytometry assay.

### OVA peptide pulse

BMDCs were incubated with 200 ng/ml LPS or 0.01 mg/ml MSA-2 for one day. Then, cells were pulsed with 50 μM OVA peptide SIINFEKL (RP10611, GenScript) for six hours [31]. Cells were harvested afterward, washed two times, and stained for flow cytometry. Abs targeting CD11c (553,801, BD) and H-2 Kb-SIINFEKL complex (141,606, BioLegend) were used in the flow cytometry assay.

### Murine tumor models

We explored the antitumor activity of the combination of MSA-2 and YM101 in multiple types of syngeneic tumors. MSA-2 was administrated orally with a single dose of 50 mg/kg. Mice received equivalent mole Ab treatment on alternate days three times (6.6 mg/kg Isotype; 6.6 mg/kg α-PD-L1; and 8.9 mg/kg YM101). Tumor volume was measured on alternate days. Mice were euthanatized when tumor volume was larger than 2500 mm^3^ or when the experiment ended.

For the subcutaneous tumors, 1 × 10^6^ CT26, 3 × 10^6^ H22, and 2 × 10^5^ B16 cells were implanted in the right groin of BALB/c and C57BL/6 mice. For the orthotopic breast cancer model, 1 × 10^5^ EMT-6 cells were injected into the right mammary fat pad of BALB/c mice. All tumor-carrying mice were randomly divided into six groups: control, MSA-2, α-PD-L1, MSA-2 combined with α-PD-L1, YM101, and MSA-2 combined with YM101 (8 mice per group). Treatment was started when tumor volume reached 50 ~ 100 mm^3^. For the rechallenge model, BALB/c mice were subcutaneously inoculated with 1 × 10^6^ CT26 cells. Two weeks after the last dose of Ab injection, the untreated or tumor-free mice (tumor shrinks beyond measurement) were rechallenged with 1 × 10^6^ CT26 cells.

### Intratumoral cytokine and chemokine detection

BALB/c mice were challenged with 1 × 10^5^ EMT-6 cells. Tumor-bearing mice were treated with a single dose of 50 mg/kg MSA-2 when tumor volume reached 300 mm^3^. Six hours after MSA-2 treatment, mice were killed and tumors were collected for intratumoral cytokine and chemokine detection. The gross protein level of tumor tissue homogenate was measured by BCA protein assay kit (P0010S, Beyotime). The concentrations of cytokines and chemokines in the homogenate were detected by ELISA (MIFNB0, R&D) and multiplex fluorescence-encoded beads (741,044, BioLegend; 740,451, BioLegend).

### Flow cytometry for tumor immune profiling

Minced tumors were digested with the dissociation buffer containing 1 mg/ml Collagenase B (11,088,807,001, Roche), 0.5 mg/ml DNase I (abs47047435, Absin), and 0.5 mg/ml Hyaluronidase (H3884, Sigma-Aldrich) at 37 °C for 40 min. Then, the suspension was filtered, and the centrifuged cells were treated with red blood cell lysis buffer (C3702, Beyotime). Subsequently, the cells were labeled with Fixable Viability Stain 780 (565,388, BD) and blocked with α-CD16/32 (101,339, BioLegend). Staining reagents used in flow cytometry included Abs targeting CD45 (560,510, BD), CD3e (562,600, BD), CD8α (563,068, BD), CD49b (740,363, BD), CD44 (561,859, BD), CD62L (553,152, BD), CD69 (566,500, BD), CD25 (553,075, BD), Ki67 (556,027, BD), Perforin (11-9392-82, ThermoFisher), Granzyme-B (372,204, BioLegend), TNF-α (563,943, BD), IFN-γ (560,660, BD), CD11c (566,504, BD), CD80 (560,016, BD), CD86 (561,962, BD), CD11b(101,206, BioLegend), F4/80 (565,411, BD), CD206 (141,708, BioLegend), I-A/I-E (107,608, BioLegend). Auxiliary reagents used in flow cytometry included Brilliant Stain Buffer (563,794, BD), GolgiPlug (555,029, BD), and FOXP3 Fix/Perm Buffer Set (421,403, BioLegend). Cell number per 100 mg tumor tissue was measured by Vi-Cell Auto (Beckman). All flow cytometry assays were conducted by FACSCelesta (BD) and analyzed by FlowJo (BD).

### Immunofluorescent (IF) and immunohistochemistry (IHC) staining

Tissues were fixed with 10% formaldehyde solution for two days. Subsequently, tissues are embedded in paraffin wax and transferred to the slice. IF staining was performed in the tyramide signal amplification method [32]. The Abs targeting PCNA (BM0104, Boster), Ki67 (ab16667, Abcam), CD8 (ab217344, Abcam), and CD4 (ab183685, Abcam) were used in the assays. Tunel (C1086, Beyotime) and picrosirius red staining (365,548, Sigma-Aldrich) assays were performed following manufacturers’ recommendations. Captured images were overviewed using Caseviewer or Hamamatsu Nanozoomer software, and two pathologists defined the regions of interest (ROIs).

The staining was quantitatively evaluated using ImageJ 1.53 (NIH). The mean infiltration depth of CD4^+^/CD8^+^ cells was assessed as previously described [19, 22]. Briefly, the infiltration depth was measured as the minimum distance of CD4/8^+^ cells to the border and scaled by tumor radius. The levels of Ki67, PCNA, and Tunel were measured by the proportions of positive pixels. The integral optical density (IOD) of picrosirius red staining was used to measure collagen expression.

### Bulk RNA-seq

Immature BMDCs were treated with 0.01 mg/ml MSA-2 or vehicle for one day to validate the effects of MSA-2 on DC. On the next day, cells were collected for RNA-seq. Besides, to explore the effects of combination therapy on the TME, B16 tissues were harvested after treatment. The total RNA was extracted by TRIzol. RNA-seq was based on Illumina Hiseq platform (performed by Wuhan SeqHealth). The reference genome was Mus_musculus.GRCm38. Differentially expressed gene (DEG) was screened with edgeR package (version 3.36) in R software (version 4.1.2) (fold change threshold: 2.0; *p* value threshold: 0.05). For in vitro experiments, the analyses were performed as MSA-2 vs. Vehicle. For in vivo experiments, the analyses were conducted as follows: Combination vs. Vehicle, Combination vs. MSA-2, and Combination vs. YM101. Six immune signatures were used to profile the characteristics of the TME. The signatures were scored as previously described, and the scores were compared by the ROAST algorithm [22].

### Single-cell RNA-seq (scRNA-seq)

EMT-6 tissues were minced and digested with the dissociation buffer containing 1 mg/ml Collagenase B, 0.5 mg/ml DNase I, and 0.5 mg/ml Hyaluronidase at 37 °C for 40 min. Then, the suspension was filtered, and the centrifuged cells were treated with red blood cell lysis buffer. To obtain adequate immune cells for scRNA-seq, we used nanobeads (480,028, BioLegend) to enrich CD45^+^ immune cells. Tumors from two different mice were pooled as one sample. Alive cells were loaded on 10 × Genomics Chromium Controller, and scRNA-seq was performed according to the manufacturer’s instructions. In this assay, 24 tumors from four groups (Control, MSA-2, YM101, and Combination; *n* = 6) were collected, eventually pooled as 12 samples for scRNA-seq.

Raw FASTQ files were processed by Cell Ranger analysis pipeline (version 6.1.2). Libraries were aligned based on GRCm39 reference genome. Secondary analyses were performed by Seurat package (version 4.0.6) in R software (version 4.1.2). Firstly, poor-quality cells were removed based on the following criteria: (1) cells with fewer than 200 or more than 7000 expressed genes; (2) cells harboring more than 10% mitochondrial gene expression. After filtering, we got 87,865 cells in total (CTL: 29,092; MSA-2: 16,039; YM101: 15,920; Combo: 26,814). Then, we performed principal component analysis (PCA) on all 87,865 transcriptomes in Seurat (50 principal components). We used dimension reduction techniques to visualize cell distance in reduced two-dimension space, including Uniform Manifold Approximation and Projection (UMAP) and t-distributed Stochastic Neighbor Embedding (t-SNE). Unsupervised cell clustering was performed at the resolution of 1.5.

The cell types were first annotated by the SingleR package (version 1.8.0) with the ImmGen reference database [33]. Then, to validate the annotations of SingleR, we calculated the levels of immune cell-specific markers based on previous studies [34, 35]. The annotations of T cell subclusters were based on previously identified markers [36, 37]. The annotations of NK cell subclusters were according to *Itgam* and *Cd27* levels [38]. Differences in the proportion of clusters among groups were compared by chi-squared test. Then, we depicted the features of T cells, NK cells, and cDCs in different groups by MSigDB hallmark gene sets (H). The signaling enrichment was scored by Gene Set Enrichment Analysis (GSEA) with singleseqgset package (version 1.2.9) [39]. The DEGs between different groups were identified by *FindMarkers* function in Seurat (minimum detection rate = 0.1, log fold change > 0.25, adjusted *p* < 0.05). The functional enrichment analysis was performed by ClusterProfiler (version 4.2.1) [40].

### Statistical analyses

Statistical analyses were performed using GraphPad Prism (version 8.4.3) and R software. For the data fitting the normal distribution, Student’s *t* test or Welch’s correction was used for the comparison of two variables. For non-normal distribution data, Mann–Whitney test was adopted for the comparison of two variables. Statistics analysis in this study was two-sided, and *p* < 0.05 was considered statistically significant.

## Results

### MSA-2 promoted the maturation of DC

**Fig. 1:**
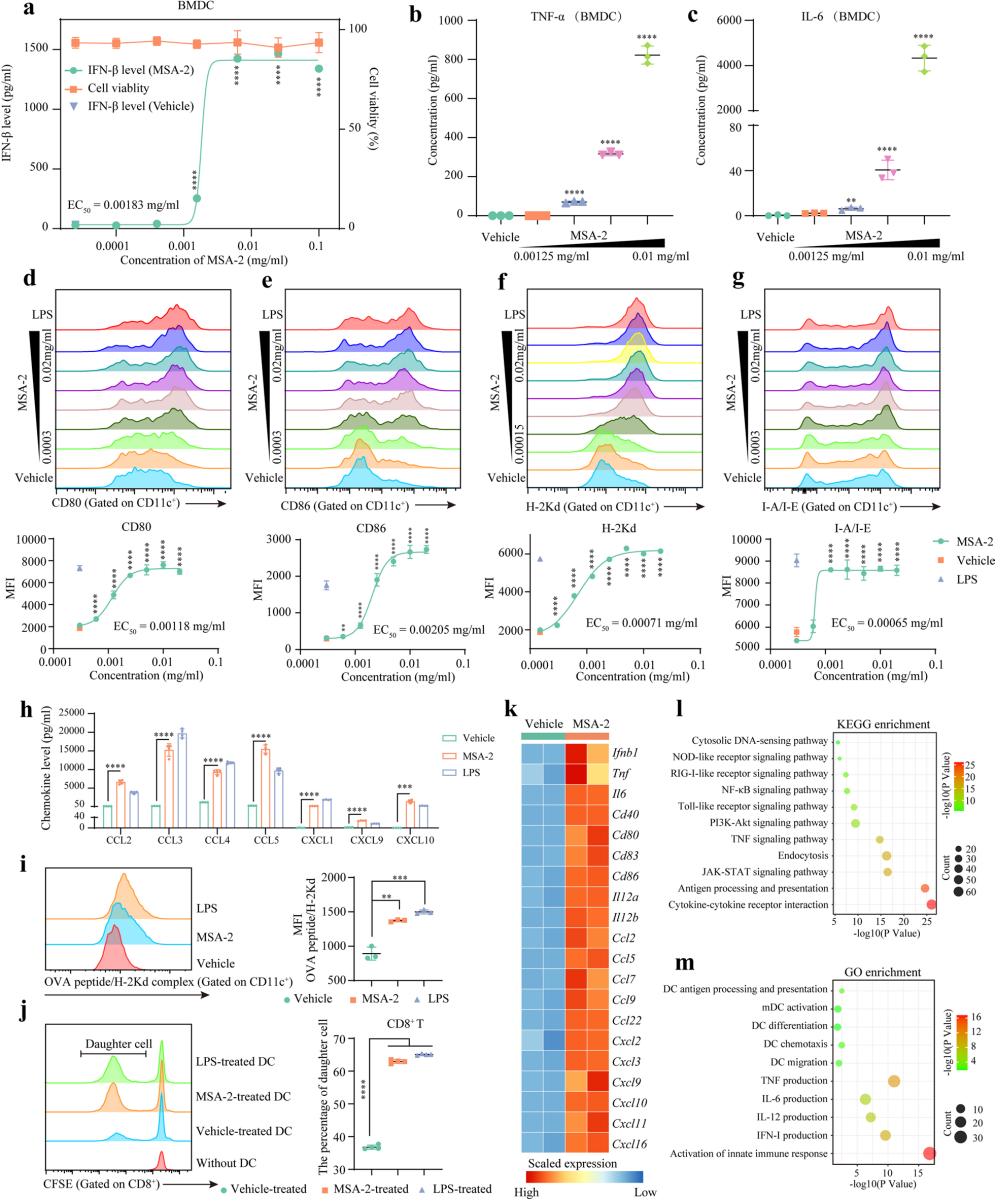
MSA-2 enhanced dendritic cell (DC) maturation. **a–c** STING pathway-associated cytokine detection. Immature bone marrow-derived DCs (BMDCs) were cultured with MSA-2 for one day, and supernatants were collected for cytokine detection. IFN-β was measured by ELISA assays; TNF-α and IL-6 were determined by multiplex fluorescence-encoded beads; and cell viability was assessed by AO/PI staining. **d–g** FACS for DC maturation markers. Immature BMDCs were cultured with MSA-2 for one day and collected for flow cytometry. MSA-2 increased CD80, CD86, H-2Kd (MHC-I), I-A/I-E (MHC-II) dose-dependent. **h** Multiplex fluorescence-encoded beads for proinflammatory chemokine detection. Immature BMDCs were cultured with MSA-2 for one day, and supernatants were collected for cytokine detection. **i** OVA peptide-plus to evaluate antigen presentation capability. Immature BMDCs were treated LPS or MSA-2 for one day. Then, cells were pulsed with OVA peptide SIINFEKL for six hours and collected for flow cytometry. **j** One-way mixed lymphocyte reaction (MLR). Stimulating cells were BMDCs derived from BALB/c mice, while responding cells were spleen cells from C57BL/6 in the MLR assays. The mixed cells (the ratio of stimulator to responder = 1:2) were cultured for four days. On day 5, the supernatants and mixed cells were collected for CFSE dilution assay. **k–m** RNA-seq assay revealing the effect of MSA-2 on BMDC. The heatmap presenting the level of genes encoding cytokines and chemokines. GO and KEGG enrichment analysis showing the pathways or biological processes significantly enriched in MSA-2-treated BMDC. *p < 0.05 means the significant difference compared to the vehicle

The dose-dependent effect of MSA-2 on STING signaling in BMDCs was evaluated by IFN-β level. We found 0.01 mg/ml MSA-2 optimally activated STING pathway (EC50 = 0.00183 mg/ml) without observable cytotoxicity (Fig. 1a). Other STING pathway-associated cytokines, including IL-6 and TNF-α, were increased after MSA-2 treatment (Fig. 1b and c). Then, we detected the effect of MSA-2 on DC maturation. The results showed that MSA-2 significantly upregulated DC maturation markers including CD80 (EC50 = 0.00118 mg/ml), CD86 (EC50 = 0.00205 mg/ml), H-2Kd (EC50 = 0.00071 mg/ml), and I-A/I-E (EC50 = 0.00065 mg/ml) in a dose-dependent manner (Fig. 1d–g). Moreover, MSA-2 promoted BMDC to secret chemokines, demonstrating the improved BMDC activity (Fig. 1h). The data of OVA peptide pulse demonstrated that MSA-2 increased the SIINFEKL/H-2Kd complex on BMDCs, indicating the enhanced peptide loading and antigen presentation capability (Fig. 1i). Parallelly, in the one-way MLR, MSA-2-treated BMDCs had a stronger ability to trigger naïve T cells proliferation (Fig. 1j) (Additional file 1: Fig S1).

To further investigate the effects of MSA-2 on BMDCs, we performed RNA-seq assays. The mRNA levels of downstream cytokines of STING pathway, DC maturation markers, and chemokines were higher in MSA-2-treated BMDCs (Fig. 1k). KEGG enrichment analysis showed cytosolic DNA-sensing, NOD-like receptor, RIG-I-like receptor, NF-κB, Toll-like receptor, PI3K-Akt, TNF, endocytosis, antigen processing and presentation, cytokine–cytokine receptor interaction pathways were enriched in MSA-2-treated BMDCs (Fig. 1l). GO enrichment analysis showed DC antigen presentation, activation, differentiation, chemotaxis, migration, cytokine production, innate immunity activation processes were enriched in MSA-2-treated BMDCs (Fig. 1m).

### YM101 synergized with MSA-2 in one-way MLR assay

**Fig. 2:**
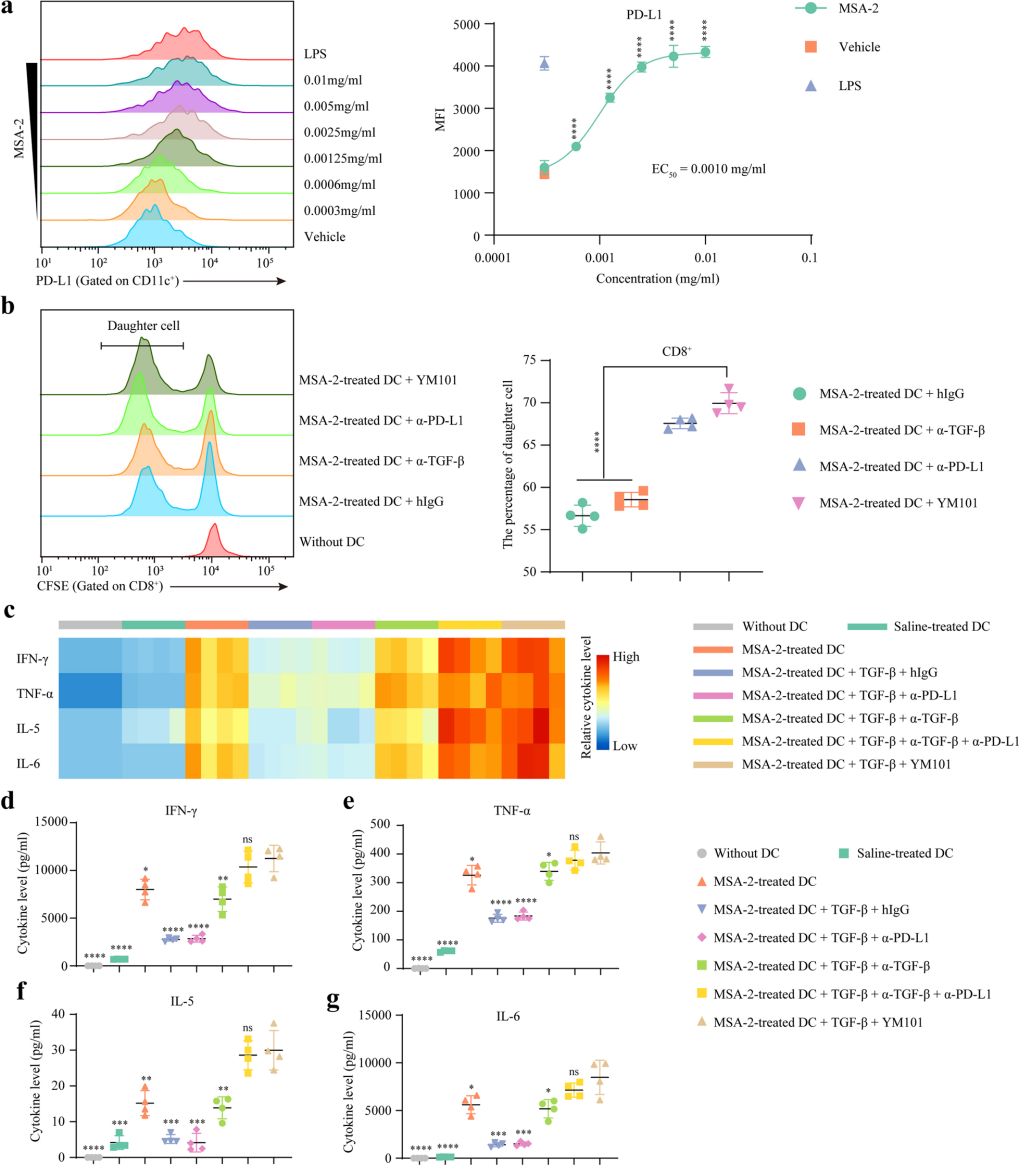
YM101 synergized with MSA-2 in one-way MLR assay. **a** FACS for PD-L1 expression. Immature BMDCs were cultured with MSA-2 for one day and collected for flow cytometry. **b** One-way mixed lymphocyte reaction (MLR). Stimulating cells were BMDCs derived from BALB/c mice, while responding cells were spleen cells from C57BL/6 in the MLR assays. The mixed cells with YM101 or controls were cultured for four days. On day 5, cells were collected for CFSE dilution assay. **c–g** The synergistic effect between MSA-2 and YM101 in the MLR system with exogenous TGF-β1. The mixed cells with TGF-β1 and antibodies for four days. On day 5, the levels of cytokines in the supernatant were detected. The heatmap shows the scaled level of cytokines, and the dot plots present the quantitative values. *p < 0.05 means the significant difference compared to the vehicle (for **a** and **b**) or MSA-2 combined with YM101 (for **d–g**)

Besides enhancing the maturation and functions of BMDCs, MSA-2 also increased PD-L1 expression in a dose-dependent manner (EC50 = 0.0010 mg/ml) (Fig. 2a). Additional α-PD-L1 or YM101 enhanced mature BMDC to induce naïve T cell proliferation (Fig. 2b) (Additional file 1: Fig S2). Our previous study reported TGF-β disturbed the cytokine release of T cell [22]. Hereto, we mimicked a high TGF-β TME by additional TGF-β1 (20 ng/ml) in the one-way MLR assay. In this system, cytokine secretion (IFN-γ, TNF-α, IL-5, and IL-6) was enhanced by MSA-2 pretreatment but hampered by exogenous TGF-β1 (Fig. 2c–g). This TGF-β1-induced weakening was restored by TGF-β blockade, while YM101 or α-TGF-β combined with α-PD-L1 further strengthened the effect of α-TGF-β. Our results indicated MSA-2 synergized with YM101 in promoting T cell activation in this high TGF-β system.

### MSA-2 regulated macrophage polarization

**Fig. 3:**
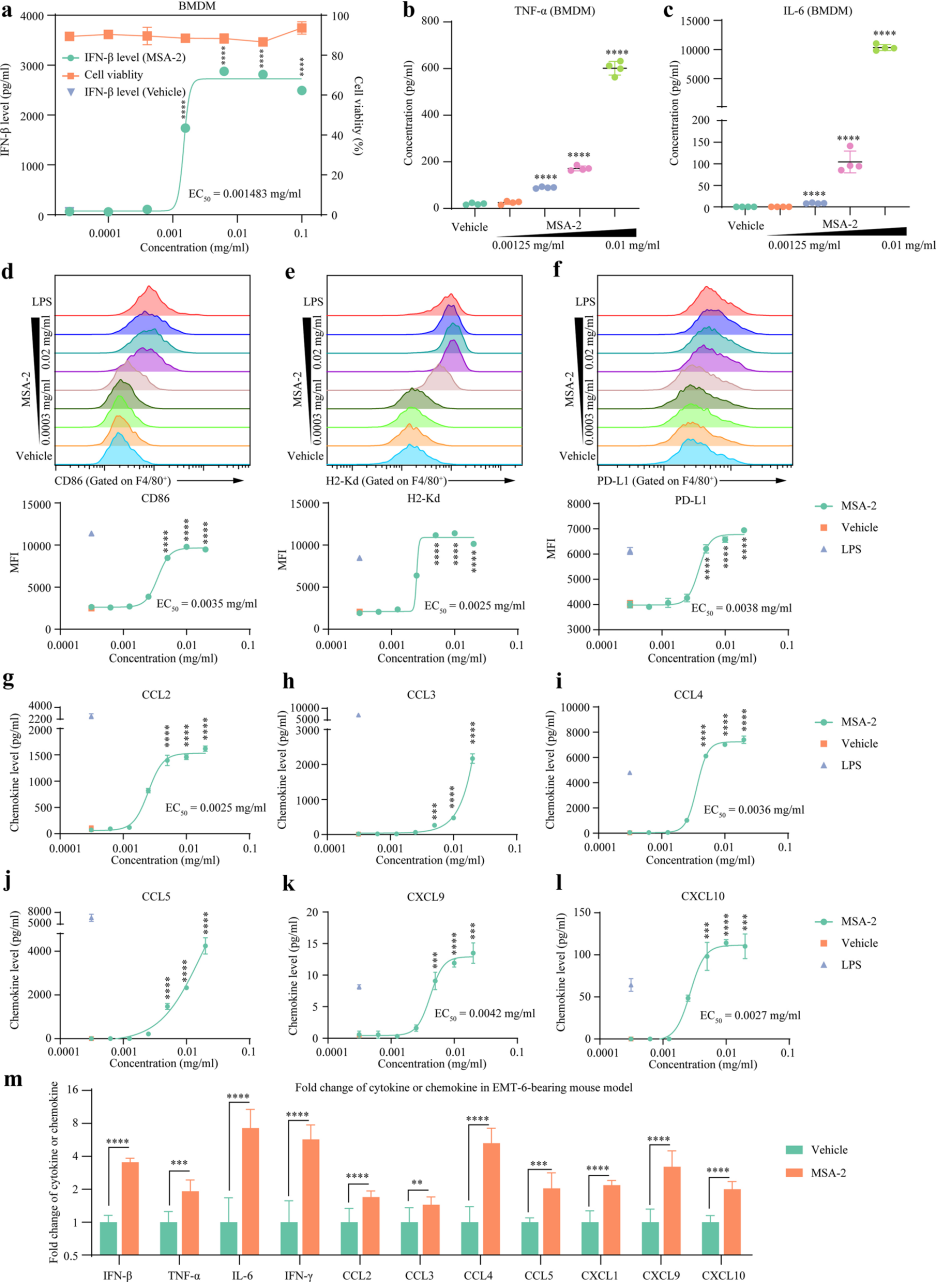
MSA-2 promoted classical activation of macrophage and altered the cytokine/chemokine panel in the TME. **a–c** STING pathway-associated cytokine detection. Unactivated bone marrow-derived macrophages (BMDMs) were cultured with MSA-2 for one day, and the levels of cytokines in the supernatant were detected. IFN-β was measured by ELISA assays; TNF-α and IL-6 were determined by multiplex fluorescence-encoded beads; and cell viability was assessed by AO/PI staining. **d–e** FACS for classical activation markers. Unactivated BMDMs were cultured with MSA-2 for one day and collected for CD86 and H-2Kd detection. **f** FACS for PD-L1 detection. **g–l** Multiplex fluorescence-encoded beads for proinflammatory chemokine detection. Unactivated BMDMs were treated with MSA-2 for one day, and supernatants were collected for cytokine detection. **m** Tumor-bearing mice were treated with a single dose of 50 mg/kg MSA-2 when tumor volume reached 300 mm^3^. Six hours after MSA-2 treatment, tumors were collected for intratumoral cytokine and chemokine detection. *p < 0.05 means the significant difference compared to the vehicle

The dose-dependent effect of MSA-2 on STING signaling in BMDMs was evaluated using IFN-β level. We observed 0.01 mg/ml MSA-2 optimally stimulated IFN-β production (EC50 = 0.00148 mg/ml) (Fig. 3a). Besides, MSA-2 increased IL-6 and TNF-α (Fig. 3b and c). To explore the effect of MSA-2 on macrophage polarization, we detected the makers of classical activation (M1-like) or alternative activation (M2-like) on BMDM after MSA-2 treatment. The results showed that MSA-2 significantly increased M1-like markers (CD86, EC50 = 0.0035 mg/ml; H-2Kd, EC50 = 0.0025 mg/ml) (Fig. 3d and e) but without obvious effects on CD80, I-A/I-E, and M2-like marker CD206 (Additional file 3: Figure S3a-S3c). Besides, MSA-2 upregulated PD-L1 on BMDMs in a dose-dependent manner (EC50 = 0.0038 mg/ml) (Fig. 3f). Moreover, some classical activation-associated chemokines such as CCL2, CCL3, CCL4, CCL5, CXCL9, and CXCL10 were increased after MSA-2 treatments (Fig. 3g–l). Additional TGF-β had no obvious effects on M1-like or M2-like markers (Additional file 3: Figure S3d-S3g) and chemokines (Additional file 4: Figure S4).

### MSA-2 upregulated cytokines and chemokines in the TME

Considering the effects of MSA-2 on cytokine and chemokine production in DC and macrophage, we explored the intratumoral cytokine and chemokine alterations after MSA-2 treatment in vivo. Six hours after oral MSA-2 treatment, IFN-β, IL-6, TNF-α, IFN-γ, and classical activation-associated chemokines including CCL2, CCL3, CCL4, CCL5, CXCL1, CXCL9, and CXCL10 were markedly upregulated in EMT-6 tissues (Fig. 3m). Further in vitro exploration indicated that the effect of MSA-2 on STING signaling was much weaker in cancer cells than in BMDCs and BMDMs (Additional file 5: Figure S5). This difference meant immune cells might contribute more than tumor cells to intratumoral cytokine and chemokine alterations.

### The antitumor effect of combination therapy

**Fig. 4:**
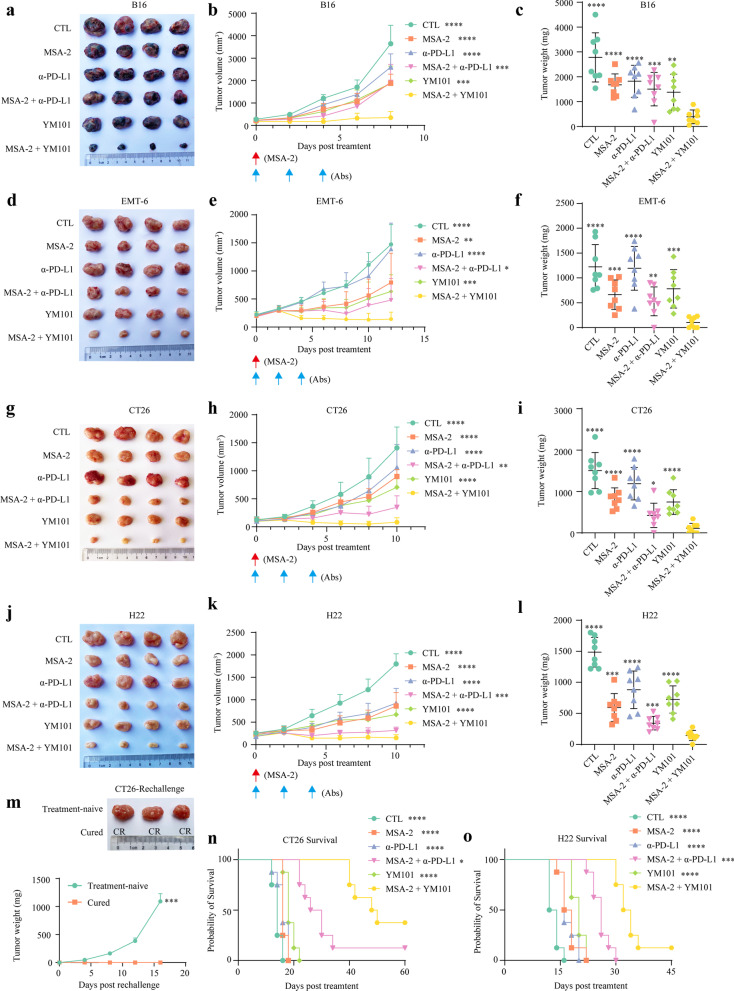
The antitumor activity of MSA-2 plus YM101 in murine models. MSA-2 was administrated orally with a single dose of 50 mg/kg. Mice received equivalent mole Ab treatment on alternate days three times (6.6 mg/kg Isotype; 6.6 mg/kg α-PD-L1; and 8.9 mg/kg YM101). All tumor-carrying mice were randomly divided into six groups: control, MSA-2, α-PD-L1, MSA-2 combined with α-PD-L1, YM101, and MSA-2 combined with YM101 (8 mice per group). Red arrow refers to MSA-2 treatment, and blue arrow refers to antibodies. Tumor volume was measured on alternate days. Mice were euthanatized when tumor volume was larger than 2500 mm^3^ or when the experiment ended. **a–l** For the subcutaneous tumors, 1 × 10^6^ CT26, 3 × 10^6^ H22, and 2 × 10^5^ B16 cells were implanted in the right groin of BALB/c and C57BL/6 mice. For the orthotopic breast cancer model, 1 × 10^5^ EMT-6 cells were injected into the right mammary fat pad of BALB/c mice. **m** For the rechallenge model, BALB/c mice were subcutaneously inoculated with 1 × 10^6^ CT26 cells. Two weeks after the last dose of treatment, the cured or untreated mice were rechallenged with 1 × 10^6^ CT26 cells. CR: complete regression. ***n–o*** The overall survival curves of CT26 and H22 models. *p < 0.05 means the significant difference compared to denote the significant difference relative to MSA-2 combined with YM101

We investigated the antitumor activity of the combination strategy in B16, EMT-6, CT26, and H22 models. Here, we observed that the efficacy of the combination was much better than monotherapies in low immunogenic (B16) and high TGF-β (EMT-6, CT26, and H22) models [19, 41, 42]. Besides, MSA-2 plus YM101 also had a superior in vivo activity to MSA-2 plus α-PD-L1 in these non-inflamed tumors (Fig. 4a–l). The results of the CT26 rechallenge assay showed that the combination provided long-term antitumor immunity (Fig. 4m). Additionally, we explored the effect of the combination therapy on tumor-bearing mouse survival. MSA-2 combined with YM101 treatment prolonged survival, superior to MSA-2 plus α-PD-L1 and monotherapies (Fig. 4n and Fig. 4o).

### The combination treatment reshaped the tumor immune profile

**Fig. 5:**
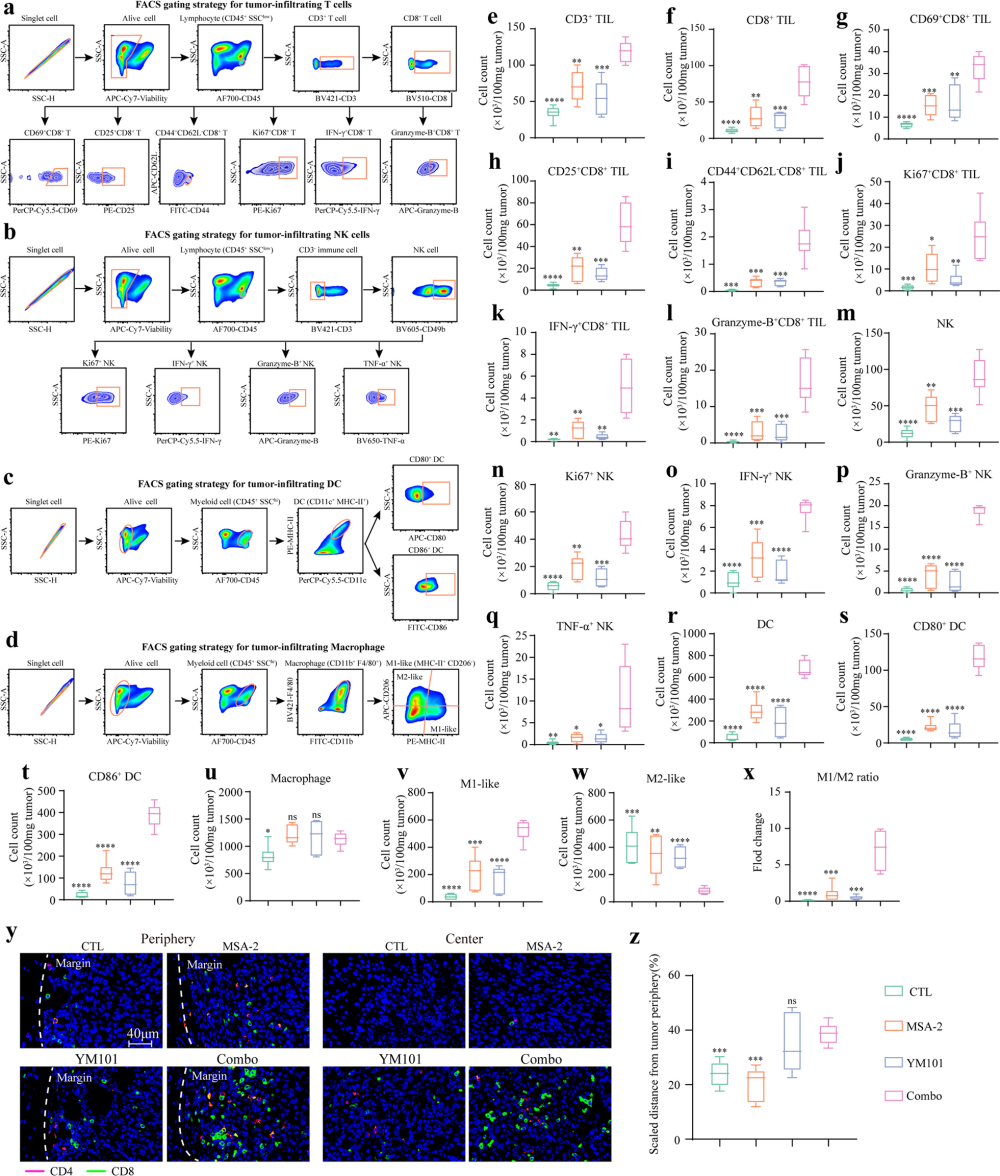
MSA-2 combined with YM101 therapy improved the activities of tumor-infiltrating immune cells in EMT-6 model. **a–d** FACS gating strategies for tumor-infiltrating T, NK, DC, and macrophages. **e–x** In the FACS assays, quantification of CD3^+^ T, CD8^+^ T, activated CD8^+^ T (CD25^+^ or CD69^+^), effector memory CD8^+^ T (CD44^+^ CD62L^−^), Ki67^+^ CD8^+^ T, IFN-γ^+^ CD8^+^ T, Granzyme-B^+^ CD8^+^ T, NK, Ki67^+^ NK, IFN-γ^+^ NK, Granzyme-B^+^ NK, TNF-α^+^ NK, DC, mature DC (CD80^+^ or CD86^+^), macrophage, M1-like macrophage (MHC-II^+^ CD206^−^), and M2-like macrophage (MHC-II^−^ CD206^+^). Numbers of immune cells per 100 mg EMT-6 tissue were calculated and compared. **y** The representative immunofluorescent staining images of tumor-infiltrating T cells. Red refers to CD4^+^ staining, and green refers to CD8^+^ staining. **z** Quantification of infiltration depth of T cells. *p < 0.05 means the significant difference compared to MSA-2 combined with YM101

**Fig. 6:**
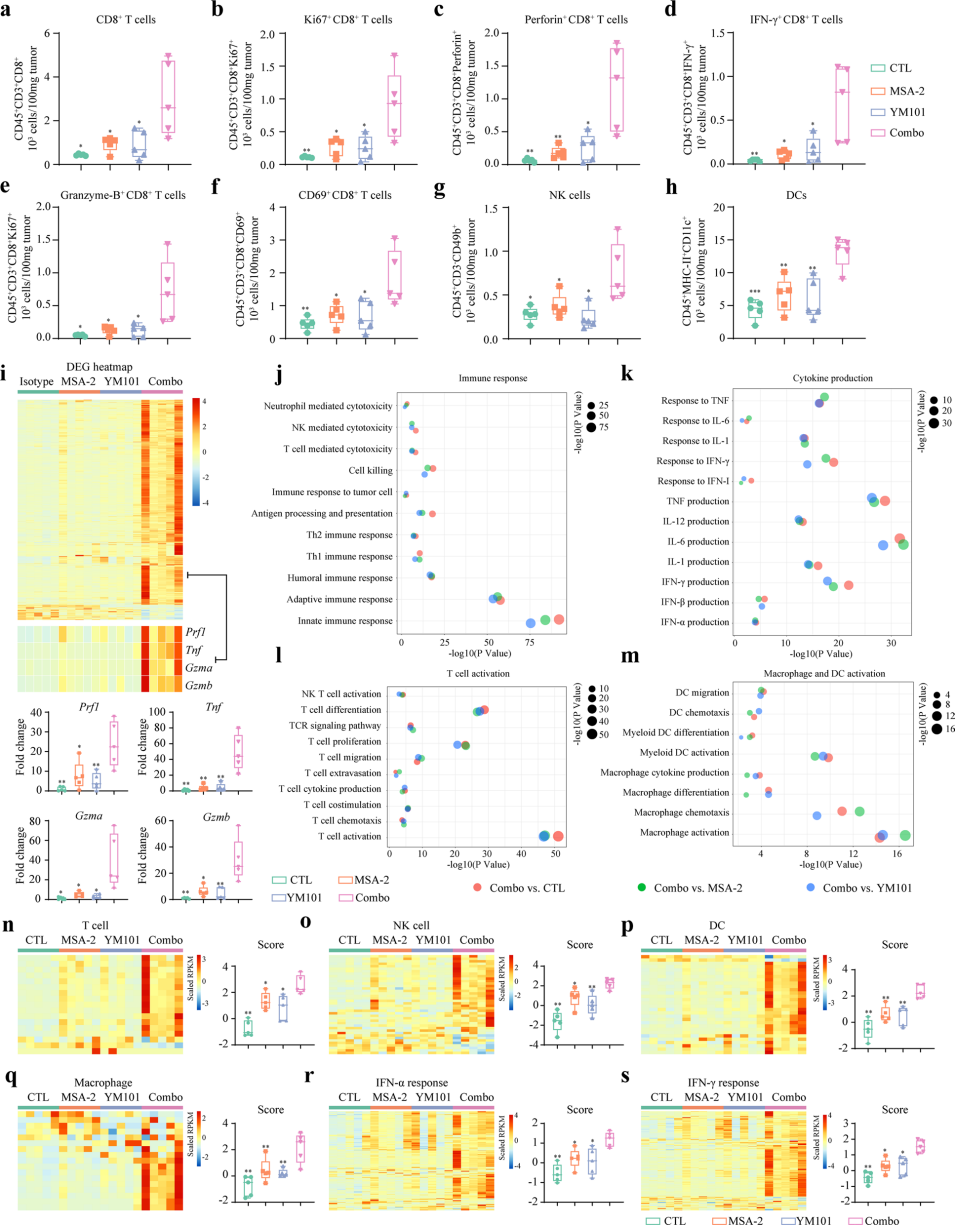
MSA-2 combined with YM101 therapy altered the TME and promoted immunity-associated gene expression in B16 model. **a–h** Quantification of CD8^+^ T cell, Ki67^+^ CD8^+^ T cell, Perforin^+^ CD8^+^ T cell, IFN-γ^+^ CD8^+^ T cell, Granzyme-B^+^ CD8^+^ T cell, CD69^+^ CD8^+^ T cell, NK cell, DC. **(i)** The heatmap shows the differentially expressed genes among four groups. The levels of immune-killing genes including *Prf1*, *Tnf*, *Gzma*, and *Gzmb* were quantitatively analyzed. **j–m** GO enrichment analysis exploring biological processes significantly enriched in MSA-2 combined with YM101 group. ***n–s*** The scores of immune signatures. The heatmaps presenting the scaled expression level of genes constituting immune signatures. *p < 0.05 means the significant difference compared to MSA-2 combined with YM101

We explored the TME alterations in immune-excluded tumors using EMT-6 model (Fig. 5a–d). Relative to the control and monotherapies, the combination therapy increased the numbers of tumor-infiltrating CD3^+^ T, CD8^+^ T, activated CD8^+^ T (CD25^+^/CD69^+^ CD8^+^), effective memory CD8^+^ T (Tem) (CD44^+^ CD62L^−^ CD8^+^), Ki67^+^ CD8^+^ T, IFN-γ^+^ CD8^+^ T, Granzyme-B^+^ CD8^+^ T, NK (CD3^−^ CD49b^+^), Ki67^+^ NK, IFN-γ^+^ NK, Granzyme-B^+^ NK, TNF-α^+^ NK, DC (CD11c^+^ MHC-II^+^), CD80^+^ DC, CD86^+^ DC, M1-like macrophage (MHC-II^+^ CD206^−^ CD11b^+^ F4/80^+^) (Fig. 5e–v). Meanwhile, the combination therapy decreased the number of M2-like macrophage (MHC-II^−^ CD206^+^ CD11b^+^ F4/80^+^) (Fig. 5w and x). Moreover, the results of IF assays showed that the combination elevated the quantity of tumor-infiltrating T cells and promoted T cell penetration into tumor center (Fig. 5y and z) (Additional file 6: Figure S6a). Picrosirius red staining indicated that combination therapy effectively decreased the peritumoral collagen deposition (Additional file 6: Figure S6b). Besides, we explored the TME changes in immune-desert tumors using B16 model (Additional file 7: Figure S7). Similarly, flow cytometry showed that the combination significantly increased the numbers of infiltrating CD8^+^ T, Ki67^+^ CD8^+^ T, Perforin^+^ CD8^+^ T, IFN-γ^+^ CD8^+^ T, Granzyme-B^+^ CD8^+^ T, CD69^+^ CD8^+^ T, NK, and DC in B16 tumors (Fig. 6a–h).

### The combination treatment enhanced the antitumor immunity-associated pathways

We conducted RNA-seq assays to investigate the effects of the combination on the immune gene profile in B16 model. The combination group had a distinct gene expression pattern, with markedly elevated immune-killing genes such as *Prf1*, *Tnf*, *Gzma*, and *Gzmb* (Fig. 6i). GO enrichment analysis showed innate/adaptive immunity, Th1/Th2 immune response, antigen processing and presentation, immune cell-mediated cytotoxicity, cytokine production, T cell activation and proliferation, DC and macrophage activity were enriched in the combination group, relative to the other three groups (Fig. 6j–m). Moreover, we comprehensively evaluated the TME status with six immune signatures. The T cell, NK cell, DC, macrophage, IFN response signature scores were highest in the combination group (Fig. 6n–s) (Additional file 8: Figure S8). The bulk RNA-seq indicated that the combination treatment simultaneously triggered innate and adaptive immunity, providing a more immune-supportive TME. IF staining showed the combination therapy increased tumor apoptosis but decreased proliferation in B16 and EMT-6 models (Additional file 9: Figure S9).

### Single-cell transcriptome atlas of the TME after the combination therapy

**Fig. 7:**
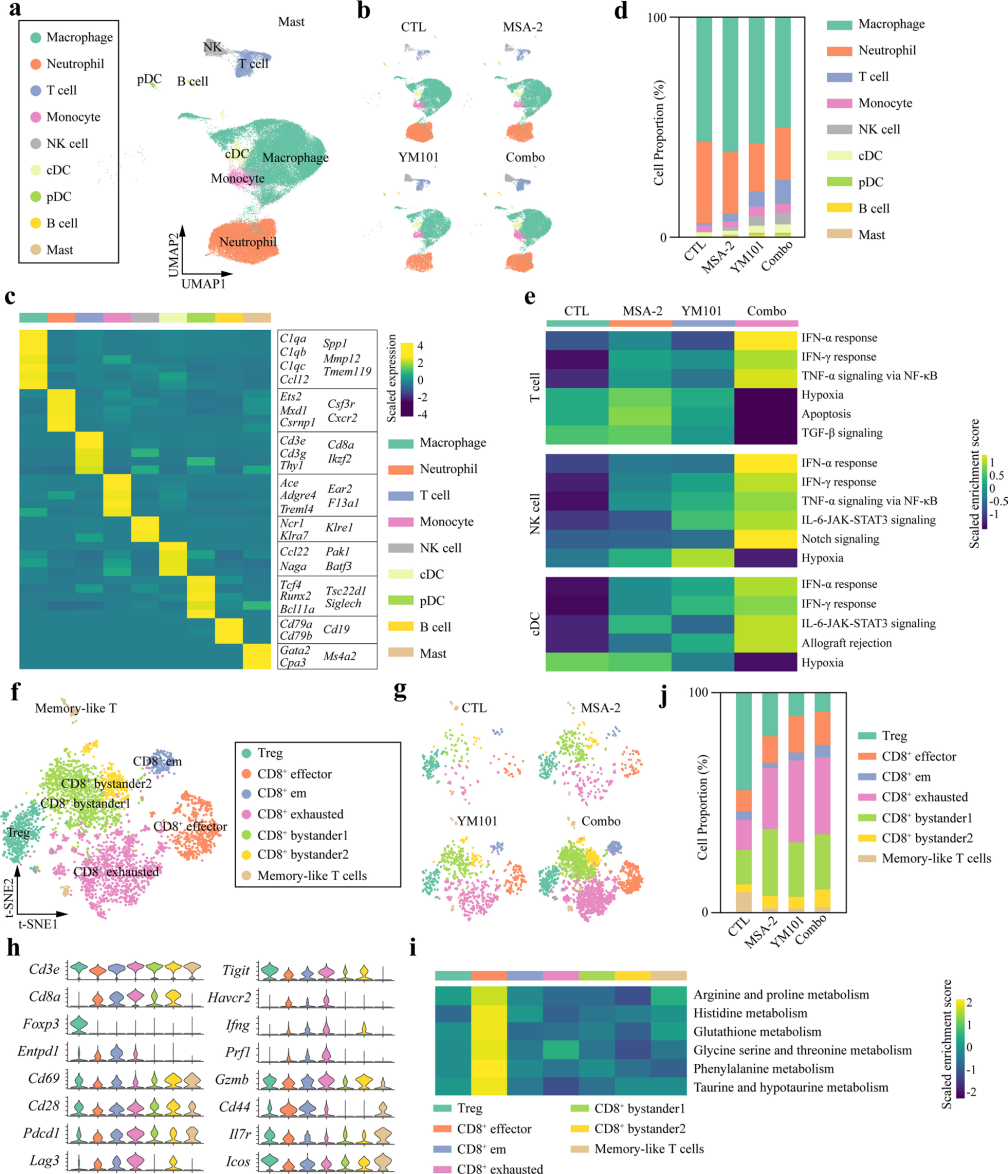
Single-cell transcriptome atlas of the TME after the combination therapy in EMT-6 model. **a** Uniform manifold approximation and projection (UMAP) plot for immune cells. **b** UMAP plot for the treatment group-specific distribution of immune cells. **c** Heatmap showing cluster-specific gene profiles. **d** Histogram representing the proportion of clusters in each group. **e** Heatmap depicting the results of GSEA using Hallmark gene sets. **f** T-distributed stochastic neighbor embedding (t-SNE) plots for tumor-infiltrating T cells. **g** t-SNE plots for the treatment group-specific distribution of T cells. **h** Violin plots showing T cell subcluster-specific gene profiles. **i** Heatmap depicting the results of GSEA using KEGG gene sets for T cell subclusters. **j** Histogram representing the proportion of T cell subclusters in each group

We conducted scRNA-seq using EMT-6 tumors to map the tumor immune transcriptome atlas comprehensively. To define major cell clusters, we performed unsupervised clustering analysis and identified 34 clusters (Additional file 10: Figure S10a). In this work, we focused on CD45^+^ immune clusters. Based on SingleR annotations and *Ptprc* level, a fraction of non-immune clusters (mainly fibroblasts or tumor cells) was excluded (Additional file 10: Figure S10b). As a result, 71,372 cells were retained, including 24,008 from the control, 13,303 from MSA-2 group, 10,086 from YM101 group, and 23,975 from the combination group. Combining SingleR annotations and the profile of cell-specific marker genes (Additional file 11: Figure S11), we classified immune clusters into nine cellular lineages (macrophage, neutrophil, T cell, monocyte, NK cell, cDC, pDC, B cell, and mast) (Fig. 7a–c) (Additional file 12: Figure S12). Notably, the proportions of tumor-infiltrating T cell (CTL: 1.0%; MSA-2: 3.4%; YM101: 6.9%; Combo: 10.7%; *χ*^2^, *p* < 0.0001), NK cell (CTL: 0.5%; MSA-2: 1.7%; YM101: 4.2%; Combo: 5.3%; *χ*^2^, *p* < 0.0001), and cDC (CTL: 1.9%; MSA-2: 1.9%; YM101: 3.3%; Combo: 3.9%; *χ*^2^, *p* < 0.0001) were increased in all treatment groups and peaked in the combination treatment group (Fig. 7d). Subsequently, we used MSigDB hallmark gene sets to present the features of T cells, NK cells, and cDCs among different groups. The results of GSEA demonstrated that IFN-α response, IFN-β response, and TNF-α signaling were enriched in T cells of the combination group. On the contrary, the enrichment scores of hypoxia, apoptosis, and TGF-β signaling were lowest in T cells of the combination group (Fig. 7e). NK cells and DCs in the combination group exhibited similar biological characteristics to T cells. Remarkably, Notch signaling was significantly enriched in NK cells of the combination group, which might contribute to higher cytolytic effector capacity [43, 44].

To further explore the alterations in single-cell transcriptome among different groups, we calculated DEGs and performed functional enrichment analysis using GO gene sets. The results showed that T cell activation, TCR signaling, IL-2 production, and leukocyte-mediated cytotoxicity were significantly enriched in T cells of the combination group (Additional file 13: Figure S13). NK cells of the combination group also had the most enriched cell killing and NK-mediated cytotoxicity. Besides, positive regulation of inflammatory response and IL-12 production were enriched in cDCs of the combination group. The single-cell transcriptome data showed that combination therapy expanded the ratio of effectors and enhanced their functions.

### Reclustering analysis of T and NK cells

Given the vital role of T cells in cancer immunology, we performed reclustering analysis of T cells and identified nine T cell subclusters. The subpopulations were annotated with classical T cell markers. Subclusters with *Entpd1* expression were assigned as tumor-specific T cells, including Treg (*CD4*^+^ *Foxp3*^+^), CD8^+^ effector (*Gzmb*^+^ *Prf1*^+^ *Ifng*^+^), CD8^+^ effector memory (*Cd44*^+^ *Cd69*^+^), CD8^+^ exhausted subclusters (*Pdcd1*^+^ *Lag3*^+^ *Tigit*^+^ *Havcr2*^+^). Subclusters without *Entpd1* expression were assigned as bystander T cells, including CD8^+^ CD69^+^ (*Cd69*^+^ *Entpd1*^*−*^) and CD8^+^ CD69^*−*^ (*Cd69*^*−*^* Entpd1*^*−*^) bystander T subclusters [37]. The rest of subcluster was assigned as memory-like T cells (*Il7r*^+^ *Icos*^+^ *Gzmb*^*−*^* Gzmk*^*−*^) (Fig. 7f–h) [36]. Relative to other T cell subclusters, CD8^+^ effector had multiple significantly enriched metabolism pathways, including arginine, proline, histidine, glutathione, glycine, serine, threonine, phenylalanine, taurine, and hypotaurine (Fig. 7i). The proportion of tumor-infiltrating Treg (CTL: 44.4%; MSA-2: 19.9%; YM101: 10.8%; Combo: 8.5%; *χ*^2^, *p* < 0.0001) of T cell was lowest in the combination treatment group (Fig. 7j). On the contrary, the ratios of tumor-infiltrating CD8^+^ effector, CD8^+^ effector memory, and CD8^+^ exhausted subpopulations to T cells were increased in YM101 and the combination treatment group.

Based on the levels of *Itgam1* and *Cd27*, NK cells were assigned as four subclusters: *Itgam1*^−^*Cd27*^*−*^, *Itgam1*^−^*Cd27*^+^, *Itgam1*^+^*Cd27*^+^, *Itgam1*^+^*Cd27*^*−*^ (Additional file 14: Figure S14a-S14c). For mature NK cells (*Itgam1*^+^*Cd27*^*−*^), there was no significant difference in the subcluster ratio among different treatment groups (Additional file 14: Figure S14d). However, *Itgam1*^+^*Cd27*^*−*^ NK cells in the combination therapy group had increased levels of *Tnf* and *Cd69* (Additional file 14: Figure S14e and S14f). GSEA showed *Itgam1*^+^*Cd27*^*−*^ subcluster in the combination therapy group had enriched NK-mediated cytotoxicity, ERBB, MAPK, JAK-STAT, TCR, and BCR signaling (Additional file 14: Figure S14g). Together, these findings suggest that combination therapy increases the antitumor potential of T and NK cells.

### Reclustering analysis of cDCs and macrophages

**Fig. 8:**
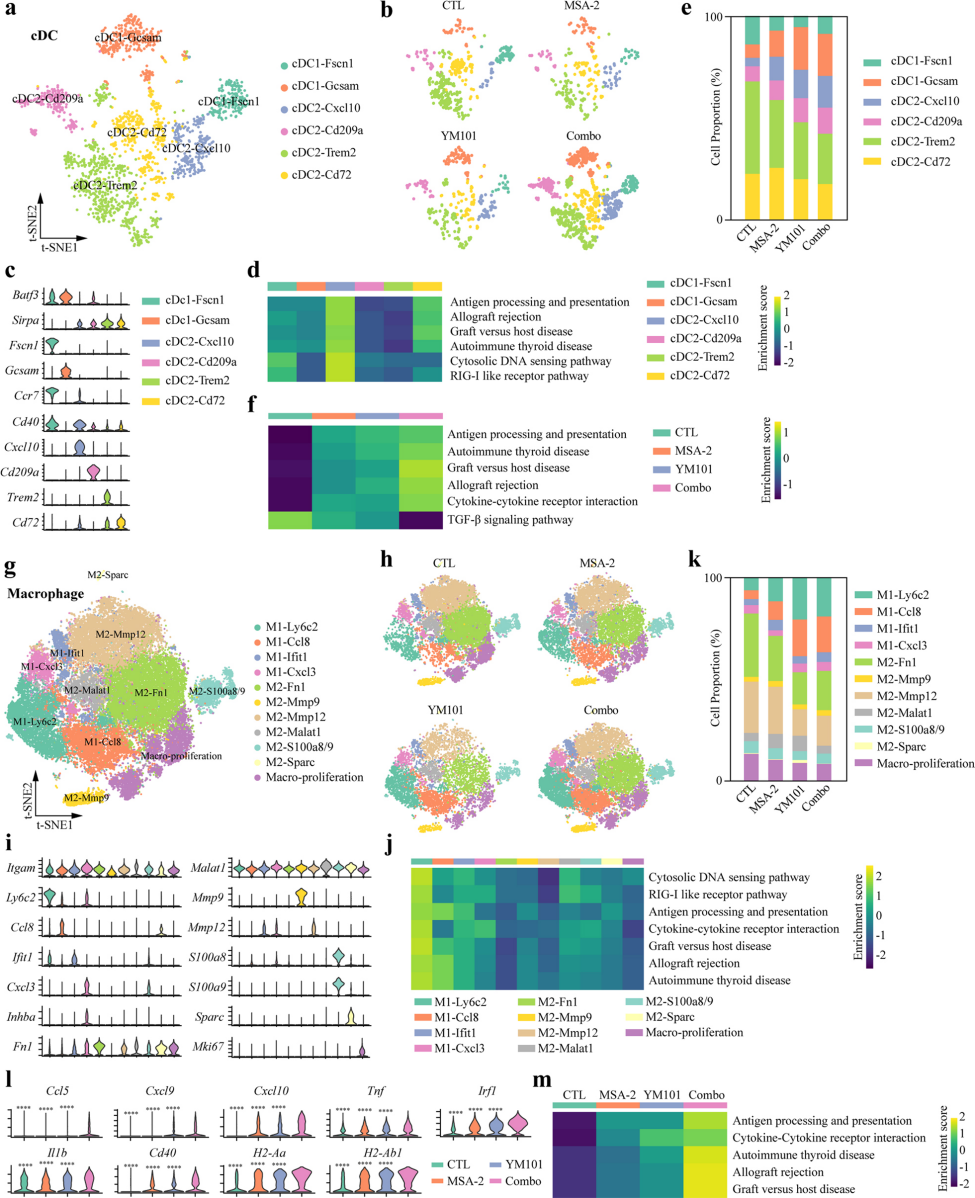
Reclustering analysis of tumor-infiltrating cDCs and macrophages. **a** T-distributed stochastic neighbor embedding (t-SNE) plot for cDCs. **b** t-SNE plot for the treatment group-specific distribution of cDC subclusters. **c** Violin plots showing cDC subcluster-specific gene profiles. **d** Heatmap depicting the results of GSEA using KEGG gene sets for cDC subclusters. **e** Histogram representing the proportion of cDC subcluster in each group. **f** Heatmap depicting the results of GSEA using KEGG gene sets for *Fcscn1*^+^ cDC1 subclusters. **g** t-SNE plot for macrophages. **h** t-SNE plot for the treatment group-specific distribution of macrophage subclusters. **i** Violin plots showing macrophage subcluster-specific gene profiles. **j** Heatmap showing the results of GSEA using KEGG gene sets for macrophage subclusters. **k** Histogram representing the proportion of macrophage subcluster in each group. **l** Violin plots showing macrophage classical activation-associated gene levels in different treatment groups. **m** Heatmap depicting the results of GSEA using KEGG gene sets for *Ly6c2*^+^ M1-like subclusters. *p < 0.05 means the significant difference compared to MSA-2 combined with YM101

Our single-cell transcriptome analysis showed in addition to T and NK cells, the fraction of cDC was increased in the combination treatment groups. Next, we sought to elucidate the effects of combination therapy on cDC populations. After reclustering, we identified two cDC1 (*Fscn1*^+^ *Batf3*^+^ and *Gcsam*^+^ *Batf3*^+^) and four cDC2 (*Cxcl10*^+^ *Sirpa*^+^*, Cd209a*^+^ *Sirpa*^+^*, Trem2*^+^ *Sirpa*^+^*, and Cd72*^+^ *Sirpa*^+^) subclusters (Fig. 8a–c). The results of GSEA showed that antigen processing and presentation, autoimmune disease-associated pathways, and cytosolic DNA-sensing pathway were obviously enriched in *Cxcl10*^+^ *Sirpa*^+^ cDC2 subcluster (Fig. 8d). In the combination treatment group, the fraction of *Cxcl10*^+^ *Sirpa*^+^ cDC2 of cDC was significantly increased (CTL: 4.2%; MSA-2: 11.6%; YM101: 14.0%; Combo: 15.6%; *χ*^2^, *p* < 0.0001) (Fig. 8e). Besides, it is well established that cDC1 effectively cross-presents cancer antigen and triggers CD8^+^ T cell activation [45]. Generally, the ratio of cDC1 was highest in the combination therapy group (CTL: 20.6%; MSA-2: 20.1%; YM101: 26.4%; Combo: 29.5%; *χ*^2^, *p* < 0.0001). Although treatments did not increase *Fscn1*^+^ *Batf3*^+^cDC1 (mature cDC1)-to-cDC ratio, the ratio of *Fscn1*^+^ *Batf3*^+^cDC1 to all immune cells was modestly increased in the combination therapy group (CTL: 0.27%; MSA-2: 0.14%; YM101: 0.18%; Combo: 0.34%; *χ*^2^, *p* = 0.0009). Notably, GSEA demonstrated antigen processing and presentation pathway was enriched in three treatment groups, especially the combination therapy group, which indicated the combination therapy-mediated optimal antigen presentation (Fig. 8f).

Although we observed no significant differences in macrophage ratio among the four groups, our in vitro and in vivo data indicated that the combination treatment might promote the classical activation of macrophages. After removing doublets, the reclustering of identified macrophages revealed 11 distinct subclusters based on transcript profiles (Fig. 8g–i). As expected, the ratio of M1-like was increased in all treatment groups, especially in YM101 and the combination treatment groups (CTL: 17.6%; MSA-2: 28.6%; YM101: 46.5%; Combo: 45.9%; *χ*^2^, *p* < 0.0001) (Fig. 8j). In all macrophage subpopulations, M1-Ly6c2 (highly expressed *Ly6c2*) was the primary M1-like subcluster, with obviously enriched cytosolic DNA-sensing pathway, RIG-like receptor pathway, antigen processing and presentation, cytokine–cytokine receptor interaction, graft versus host disease, allograft rejection, and autoimmune thyroid disease (Fig. 8k). Thus, we focused on M1-Ly6c2 subcluster, which might be preferentially targeted by the combination therapy in all macrophage subpopulations. In the four groups, M1-Ly6c2 subcluster of the combination therapy had the highest *Ccl5*, *Cxcl9*, *Cxcl10*, *Tnf*, *Irf1*, *Il1b*, *Cd40*, *H2-Aa*, *H2-Ab1* (Fig. 8l). The results of GSEA showed antigen processing and presentation, cytokine–cytokine receptor interaction, graft versus host disease, allograft rejection, and autoimmune thyroid disease were markedly enriched in M1-Ly6c2 subpopulation of the combination therapy group (Fig. 8m). Collectively, our scRNA-seq data reveal the combination group promotes the classical activation of macrophage and preferentially enhanced the activities of Ly6c2^hi^ M1-like subpopulation.

## Discussion

The non-inflamed tumor is a major challenge for cancer immunotherapy. Poor tumor immunogenicity, hampered DC maturation, suboptimal T cell priming/activation, impaired T cell infiltration, and stroma-dependent exclusion participate in immune escape and contribute to immune checkpoint resistance in non-inflamed tumors [46, 47]. Hence, for non-inflamed tumors, a combination regimen targeting different immune escape mechanisms might be required [46, 48, 49]. Although our previous work had shown the BsAb YM101 reversed CAF-mediated exclusion and relieved α-PD-L1 resistance, it exhibited modest antitumor activity in immune-desert models. In this situation, fixing defects in the early stage of cancer-immunity cycle by promoting immunogenic cell death, enhancing DC maturation, or optimal T cell priming/activation facilitates sensitizing immune-desert tumors. As the core regulator of adaptive immunity, DC not only processes and cross-presents cancer antigens but provides costimulatory signals to naïve T cells. However, in the TME, DC maturation is often undermined and insufficient to induce potent cancer-specific immunity [50]. Activating DC could motivate the following T cell response, and agents targeting DC have translational potential in combination strategies.

Besides DC, macrophage is a vital regulator for antitumor immunity and immunotherapy [51]. Due to the different developmental origins, tissue localizations, and microenvironmental cues, macrophage consists of a collection of heterogeneous populations without specific phenotype. At present, most studies on macrophage are contextualized within M1/M2 system. It is widely accepted that macrophages with high levels of TNF, iNOS, and MHC-II are anti-tumorigenic, while macrophages with high levels of IL-10, ARG1, CD206, CD163 are pro-tumorigenic [52]. Now more and more evidence indicates that macrophage has a continuum of states during activation, and it should be circumspect to use these markers to define their roles in the TME [52]. Notably, although macrophage is the primary phagocytic population in the TME, they could not migrate into draining lymph nodes without CCR7 expression [53]. Hence, macrophage in tumors possesses the limited capability to prime and activate T cell. Conversely, macrophage-mediated phagocytosis negatively influences antitumor immunity by redirecting neoantigens away from DC, reducing the release of alarmins or damage-associated molecular patterns, and suppressing the activation of their own or neighboring cells [52]. Collectively, macrophage in the TME hampers the capability of DC to present antigen and prime CD8^+^ T cell. In this assay, we promoted macrophage repolarization towards anti-tumorigenic phenotype by combination therapy. The scRNA-seq and FACS data showed that MSA-2 combined with YM101 restored the latent immunostimulatory activities of tumor macrophage, acting as primary phagocyte and professional APC in the TME.

cGAS-STING pathway is a crucial cytosolic DNA-sensing machinery in innate immunity. In the TME, tumor-derived DNA or cGAMP could trigger downstream IFN-I expression in APCs (particularly DCs), enhancing antigen presentation and cross-priming [54]. STING agonist harnesses this innate immunity signal to generate IFN-I, systemically strengthening the functions of DCs, macrophages, NK cells, and T cells. Despite the setbacks in drug development, STING has always been a hot target for cancer immunotherapy. From DMXAA and cyclic dinucleotides (CDNs) to novel agonists diABZI and MSA-2, fundamental changes have occurred in drug delivery and bioavailability [55, 56]. MSA-2 is the first publicly reported oral STING agonist, with great advantage in the ease of administration [30]. We found that MSA-2 effectively promoted the activities of DC and macrophage, enhanced antigen presentation, and induced a proinflammatory cytokine/chemokine panel in the TME. Moreover, we observed the level of IFN-β production in cancer cells was much lower than in BMDCs and BMDMs, indicating the non-tumoral populations might be the primary responders of MSA-2 treatment. Actually, in some cancer cells, epigenetic silencing and oncogenic signals lead to the inactivation of cGAS-STING pathway, participating in immune escape [57]. Despite the weak effect on IFN-β production, direct MSA-2 treatment and conditional medium from MSA-2-treated DCs increased H-2Kd on cancer cells (Additional file 15: Figure S15), which might potentiate α-PD-1/PD-L1 [58]. Our data help to understand the mechanisms of MSA-2-mediated immune stimulation.

Here we reported the combination of MSA-2 with YM101 sensitized non-inflamed tumors by boosting the innate and adaptive immune response. The combination therapy exhibited potent antitumor activity in multiple murine tumor models, superior to YM101 monotherapy and MSA-2 combined with α-PD-L1. The explorations of the TME showed the combination treatment increased the quantity of activated tumor-infiltrating lymphocytes and DCs, and promoted macrophage polarization toward M1-like phenotype. Although our previous data had confirmed YM101 monotherapy also altered the TME, the magnitude of changes in the YM101 group was much lower than in the combination group. Additional MSA-2 promoted DC maturation, reprogrammed macrophage polarization, stimulated cytokine and chemokine secretion, and enhanced T cell chemotaxis, contributing to improved antitumor activity. MSA-2 not only strengthened the effect of YM101 in immune-excluded tumors but in immune-desert tumors, indicating the great potential of the combination as a universal regimen. Notably, due to the structure of bispecific antibody, YM101 might have higher specificity for some tumors with increased TGF-β and PD-L1 expression. Additionally, YM101 has strategic advantages over the conventional dual-antibody combination therapy, especially in clinical trials with complex subgroups.

M7824 is the pioneer of the second-generation α-PD-L1 agent, targeting TGF-β and PD-L1. In the early clinical studies, M7824 showed encouraging activity in advanced solid tumors [59, 60]. However, M7824 successively failed in multiple phase II or III clinical studies. The TME is more complex than expected, and the double blockade of TGF-β and PD-L1 might not bring more benefits even in tumors with active TGF-β signaling, such as biliary tract cancer. Predictive biomarkers, patient selection, and optimal combination strategy are urgent issues for developing bifunctional α-PD-L1 agents. At the present stage, Y101D, a surrogate of YM101 targeting human PD-L1 and TGF-β, has entered into a phase I clinical trial for advanced solid tumors (NCT05028556). Sharing the same biological mechanisms with M7824, Y101D might encounter similar problems in future clinical trials. Our preclinical data showed STING agonist and α-TGF-β/PD-L1 BsAb targeted three independent and complementary pathways, providing potent and durable antitumor immune protection. This preclinical work is a fundamental study in exploring potential combination strategies of Y101D in the clinic.

## Conclusion

In summary, MSA-2 stimulates DC maturation and promotes the classical activation of macrophages. In vivo experiments indicate MSA-2 reignites immunologically cold tumors by systemically enhancing the activities of innate and adaptive immune machinery. In multiple non-inflamed models, MSA-2 synergizes with YM101 to normalize the dysregulated TME and retard tumor growth. In this work, we provide proof of concept that the combination of MSA-2 and YM101 elicits a potent antitumor immunity and durable immune protection by strengthening the activities and increasing the numbers of tumor-infiltrating DCs, macrophages, T cells, and NK cells. These data illustrate that such a strategy might overcome immunotherapy resistance and improve the performance of α-TGF-β/PD-L1 BsAb in human non-flamed tumors.

## Supplementary information

**Additional file 1.** **Figure S1**: Flow cytometry for daughter CD4+ T cell in one-way mixed lymphocyte reaction (MLR). Stimulating cells were BMDCs derived from BALB/c mice while responding cells were spleen cells from C57BL/6 in the MLR assays. The mixed cells (the ratio of stimulator to responder = 1:2) were cultured for four days. On day 5, the supernatants and mixed cells were collected for CFSE dilution assay.

**Additional file 2.** **Figure S2**: Flow cytometry for daughter CD4+ T cell in one-way mixed lymphocyte reaction (MLR). Stimulating cells were BMDCs derived from BALB/c mice while responding cells were spleen cells from C57BL/6 in the MLR assays. The mixed cells with YM101 or control antibodies were cultured for four days. On day 5, cells were collected for CFSE dilution assay.

**Additional file 3.** **Figure S3**: The effects of MSA-2 and TGF-β on M1-like or M2-like macrophage markers. (a-c) Unactivated BMDMs were cultured with MSA-2 for one day and cells were collected for CD80, I-A/I-E, and CD206 detection. (d-g) Unactivated BMDMs were treated with MSA-2 and TGF-β1 for one day, and cells were harvested for CD80, CD86, H2-Kd, and CD206 detection.

**Additional file 4.** **Figure S4**: The effects of MSA-2 and TGF-β on chemokine production in BMDM. (a-f) Unactivated BMDMs were cultured with MSA-2 for one day, and supernatants were collected for chemokine detection.

**Additional file 5.** **Figure S5**: MSA-2-stimulated IFN-β expression in cancer cells. Three cell lines B16, CT26, and EMT-6 were cultured with MSA-2 for one day, and supernatants were collected for IFN-β detection with ELISA. BMDM and BMDC were used as positive controls.

**Additional file 6.** **Figure S6**: The effect of the combination therapy on T cell infiltration and peritumoral collagen deposition in the EMT-6 model. (a) Immunofluorescent staining showing T cells in tumor margin and center. (b) Picrosirius red staining showing picrosirius red staining.

**Additional file 7.** **Figure S7**: The FACS gating strategies for the B16 model.

**Additional file 8.** **Figure S8**: MSA-2 combined with YM101 therapy promoted immunity-associated gene expression in B16 model. The heatmaps showing the change folds of genes constituting immune signatures.

**Additional file 9.** **Figure S9**: The effect of the combination therapy on proliferation and apoptosis markers in B16 and EMT-6 model. (a) Tunel staining in B16 model. (b) Ki67 staining in B16 model. (c) Tunel staining in EMT-6 model. (d) Ki67 staining in EMT-6 model. (e) PCNA staining in EMT-6 model.

**Additional file 10**. **Figure S10**. (a) T-Distributed Stochastic Neighbor Embedding (t-SNE) plot depicting clusters of all immune and non-immune cells from 24 EMT-6 tumors analyzed by 10× genomics scRNA-seq. (b) Violin plot showing expression of Ptprc (encoding CD45) in all clusters from EMT-6 tumors.

**Additional file 11.** **Figure S11**. Heatmap showing cluster-specific gene profiles. (a) Macrophage-specific gene profiles. (b) Neutrophil-specific gene profiles. (c) T cell-specific gene profiles. (d) NK cell-specific gene profiles. (e) B cell-specific gene profiles. (f) Monocyte-specific gene profiles. (g) cDC-specific gene profiles. (h) pDC-specific gene profiles. (i) Mast-specific gene profiles.

**Additional file 12.** **Figure S12**. Histogram representing the proportion of clusters in each group.

**Additional file 13**. **Figure S13**. Bubble plots showing the results of DEG functional enrichment analysis using GO gene sets in T cells, NK cells, and cDCs.

**Additional file 14.** **Figure S14**. Reclustering analysis of tumor-infiltrating NK cells. (a) T-distributed stochastic neighbor embedding (t-SNE) plot for NK cells. (b) t-SNE plot for the treatment group-specific distribution of NK cell subclusters. (c) Violin plots showing NK cell subcluster-specific gene profiles. (d) Histogram representing the proportion of NK cells subcluster in each group. (e-f) Violin plots showing the levels of Cd69 and Tnf of NK cell subclusters. (g) Heatmap depicting the results of GSEA using KEGG gene sets for Itgam+ Cd27-NK cell subclusters.

**Additional file 15.** **Figure S15**. Cross talk between cancer cells and BMDCs. BMDCs were treated with 200 ng/ml LPS or different doses of MSA-2. One day later, the supernatant was discarded, and fresh medium was added. The next day, the conditioned medium from BMDCs was used for CT26 and EMT-6 culture. A day later, cancer cells were harvested for flow cytometry assay. Abs targeting H-2Kd (742436, BD) and PD-L1 (124312, BioLegend) were used in the flow cytometry assay.

## Data Availability

The dataset generated during the current study is available from the corresponding author on reasonable request. All raw RNA-seq data are publicly available in National Center for Biotechnology Information Sequence Read Archive under accession number PRJNA837188.

## Abbreviations

PD-1: Programmed cell death protein 1
TCR: T cell receptor
TME: Tumor microenvironment
TGF-β: Transforming growth factor-beta
STING: Stimulator of interferon genes
APC: Antigen presentation cell
Treg: Regulatory T
DC: Dendritic cells
BMDC: Bone marrow-derived DC
BMDM: Bone marrow-derived macrophage
DEG: Differentially expressed gene
GM-CSF: Granulocyte–macrophage colony-stimulating factor
GSEA: Gene set enrichment analysis
LPS: Lipopolysaccharides
M-CSF: Macrophage colony-stimulating factor
EC50: Half maximal effective concentration
ELISA: Enzyme-linked immunosorbent assay
CFSE: Carboxyfluorescein diacetate succinimidyl ester
IFN: Interferon
ROI: Regions of interest
IOD: Integral optical density
DEG: Differently expressed gene
CAF: Carcinoma-associated fibroblast
CDN: Cyclic dinucleotide
