# Supplementary figures and images for: Combination of oral STING agonist MSA-2 and anti-TGF-β/PD-L1 bispecific antibody YM101: a novel immune cocktail therapy for non-inflamed tumors
Source: J Hematol Oncol. 2022 Oct 8;15:142. doi: 10.1186/s13045-022-01363-8 (PMC9548169; doi:10.1186/s13045-022-01363-8)

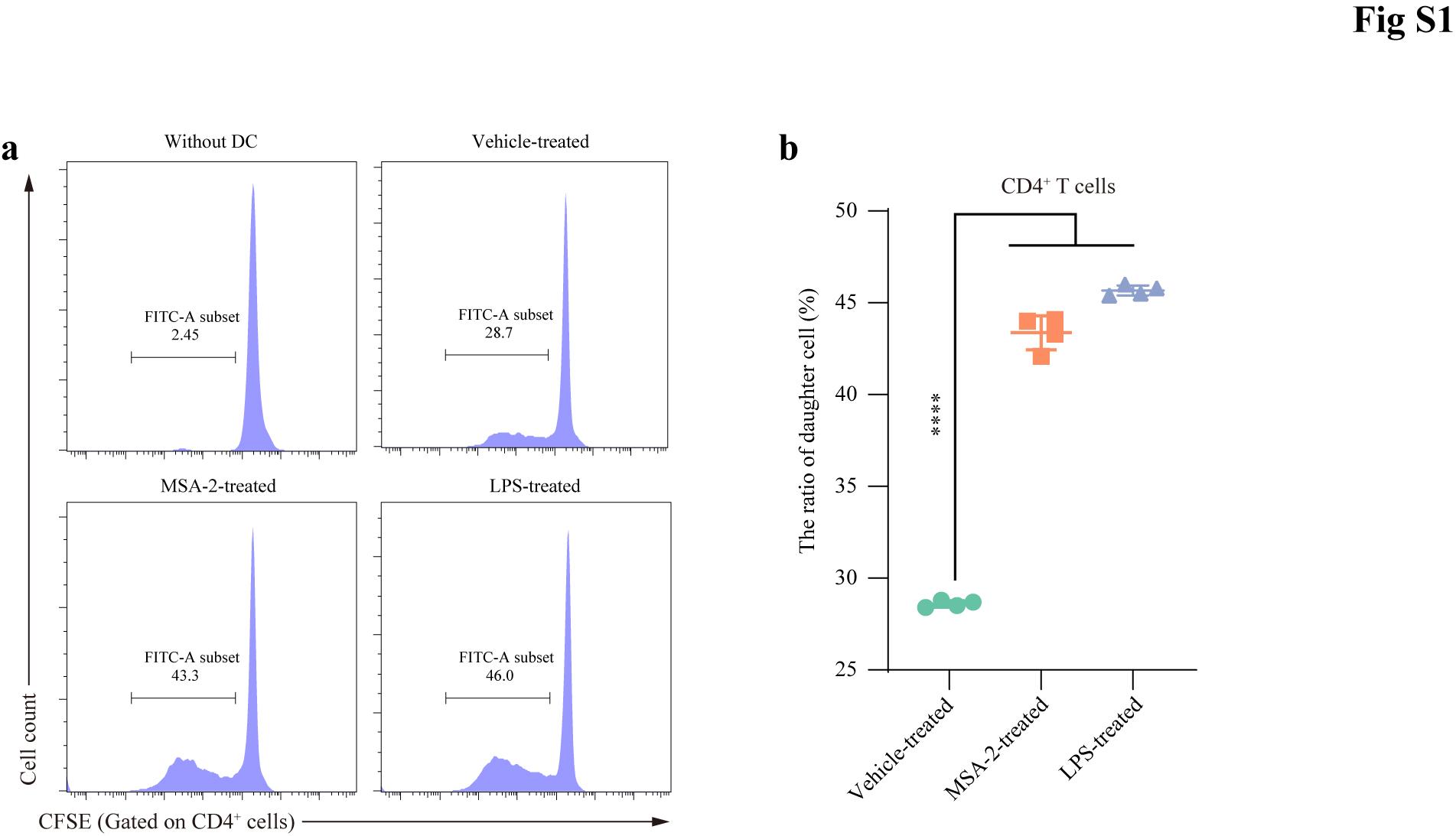

Supplement: Supplementary file 1 — Additional file 1. Figure S1: Flow cytometry for daughter CD4+ T cell in one-way mixed lymphocyte reaction (MLR). Stimulating cells were BMDCs derived from BALB/c mice while responding cells were spleen cells from C57BL/6 in the MLR assays. The mixed cells (the ratio of stimulator to responder = 1:2) were cultured for four days. On day 5, the supernatants and mixed cells were collected for CFSE dilution assay. [file 13045_2022_1363_MOESM1_ESM.jpg]

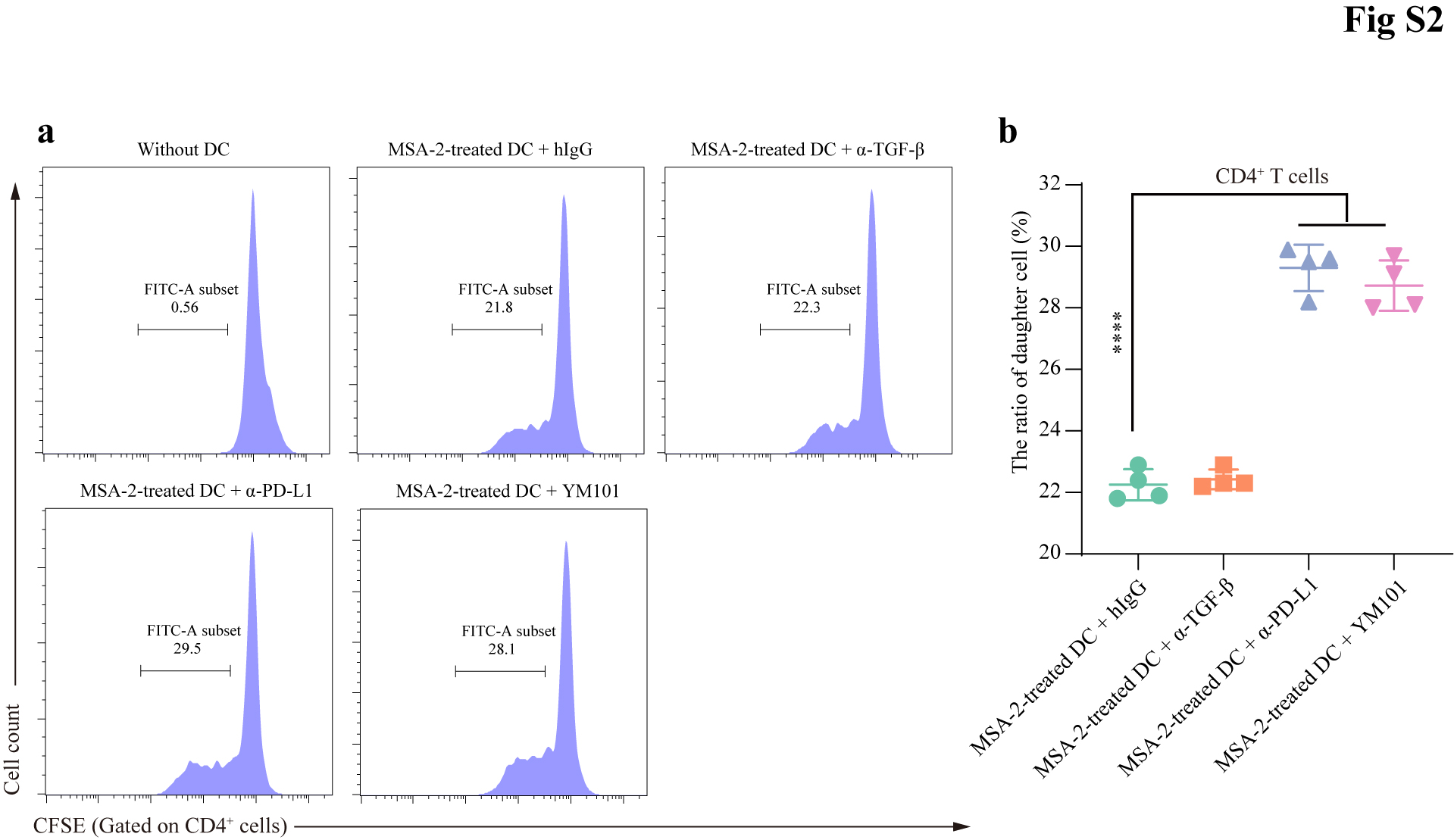

Supplement: Supplementary file 2 — Additional file 2. Figure S2: Flow cytometry for daughter CD4+ T cell in one-way mixed lymphocyte reaction (MLR). Stimulating cells were BMDCs derived from BALB/c mice while responding cells were spleen cells from C57BL/6 in the MLR assays. The mixed cells with YM101 or control antibodies were cultured for four days. On day 5, cells were collected for CFSE dilution assay. [file 13045_2022_1363_MOESM2_ESM.jpg]

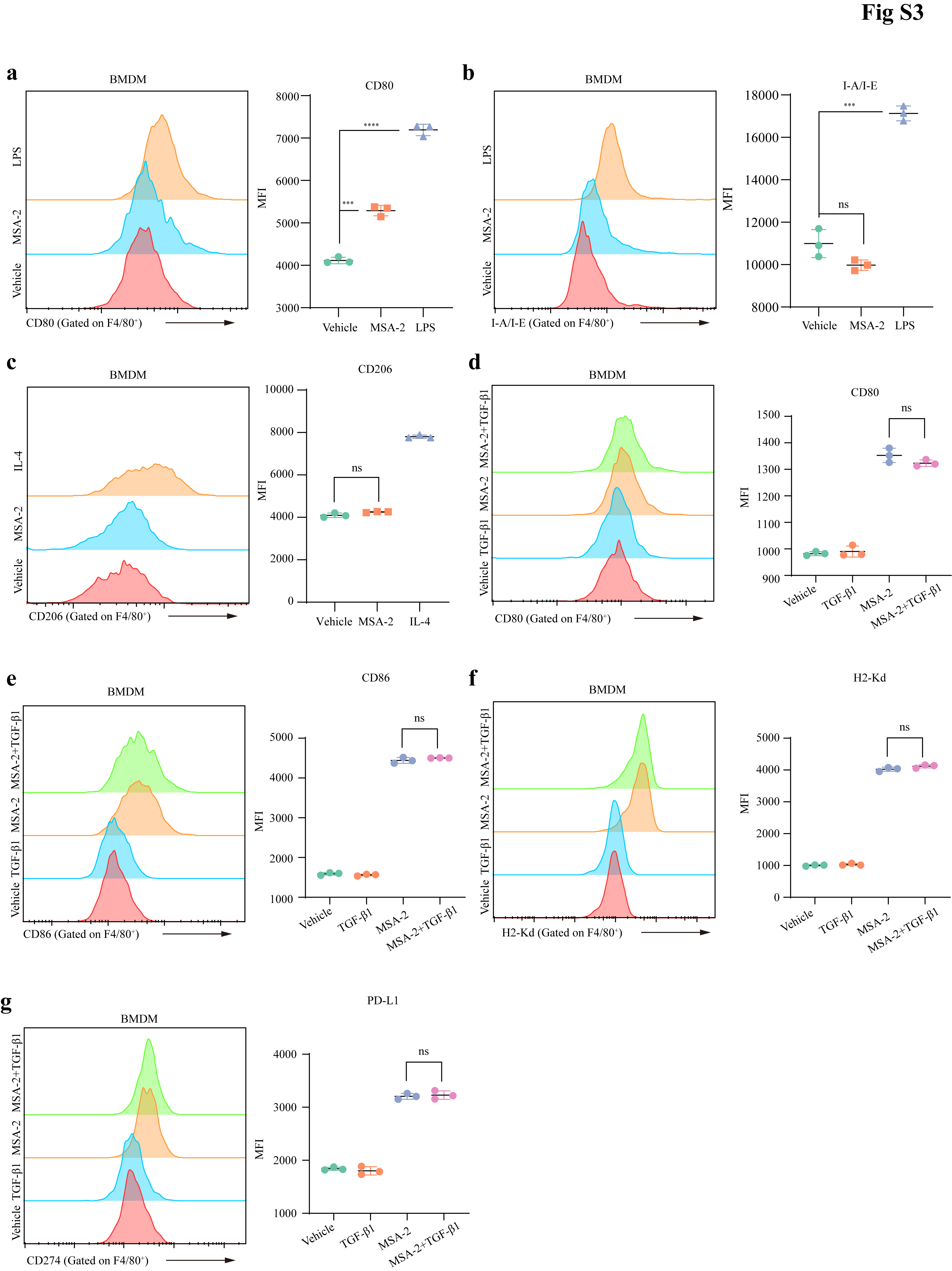

Supplement: Supplementary file 3 — Additional file 3. Figure S3: The effects of MSA-2 and TGF-β on M1-like or M2-like macrophage markers. (a-c) Unactivated BMDMs were cultured with MSA-2 for one day and cells were collected for CD80, I-A/I-E, and CD206 detection. (d-g) Unactivated BMDMs were treated with MSA-2 and TGF-β1 for one day, and cells were harvested for CD80, CD86, H2-Kd, and CD206 detection. [file 13045_2022_1363_MOESM3_ESM.jpg]

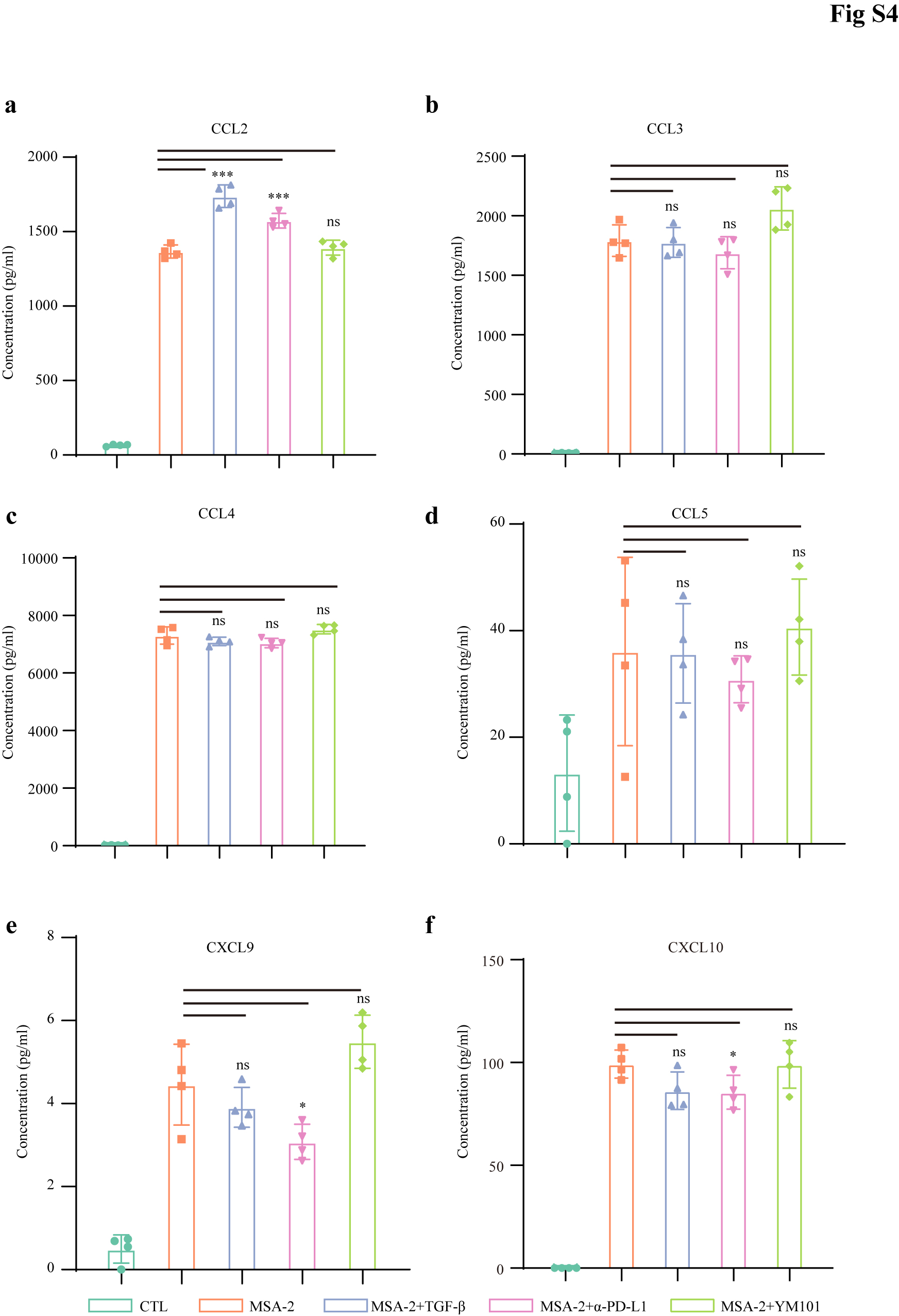

Supplement: Supplementary file 4 — Additional file 4. Figure S4: The effects of MSA-2 and TGF-β on chemokine production in BMDM. (a-f) Unactivated BMDMs were cultured with MSA-2 for one day, and supernatants were collected for chemokine detection. [file 13045_2022_1363_MOESM4_ESM.jpg]

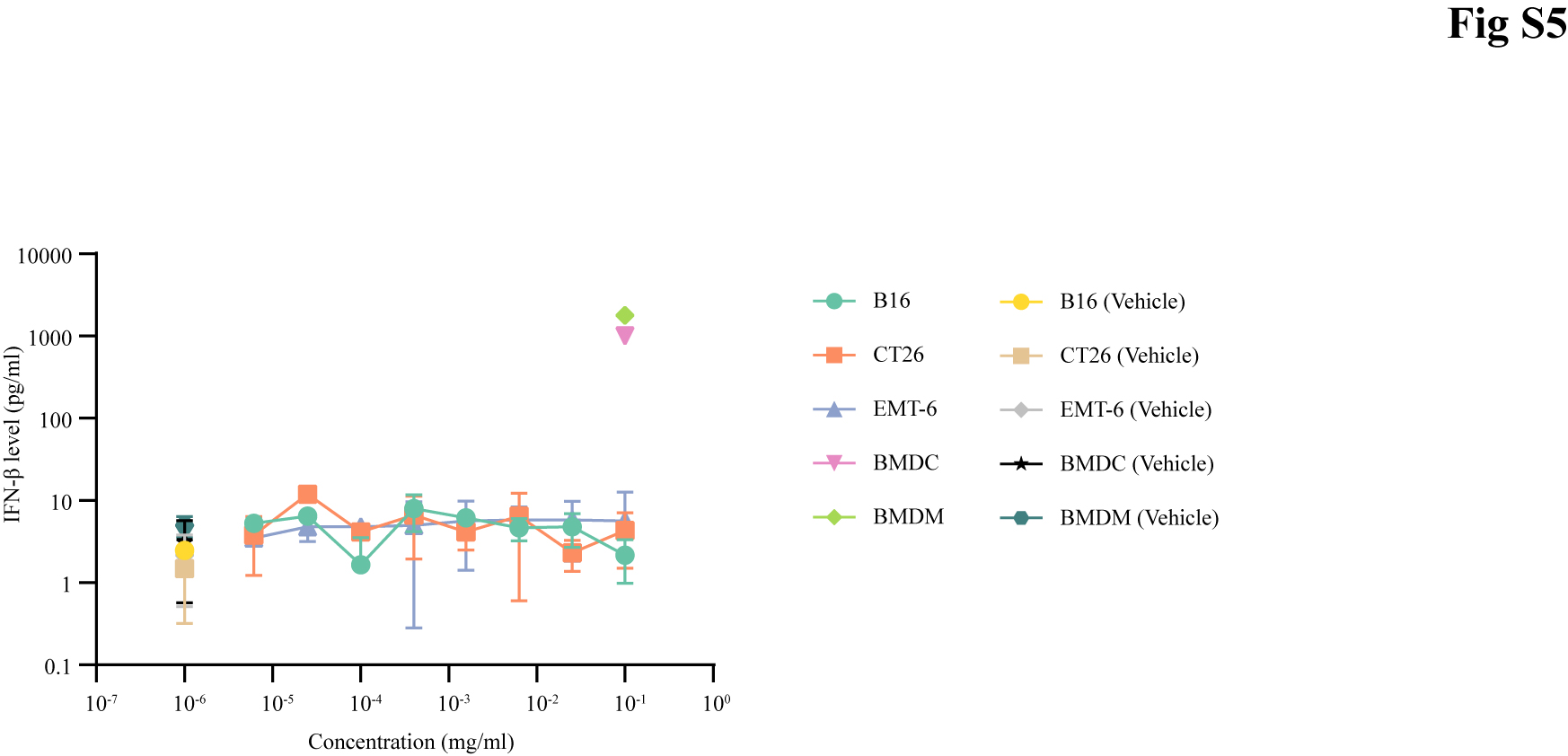

Supplement: Supplementary file 5 — Additional file 5. Figure S5: MSA-2-stimulated IFN-β expression in cancer cells. Three cell lines B16, CT26, and EMT-6 were cultured with MSA-2 for one day, and supernatants were collected for IFN-β detection with ELISA. BMDM and BMDC were used as positive controls. [file 13045_2022_1363_MOESM5_ESM.jpg]

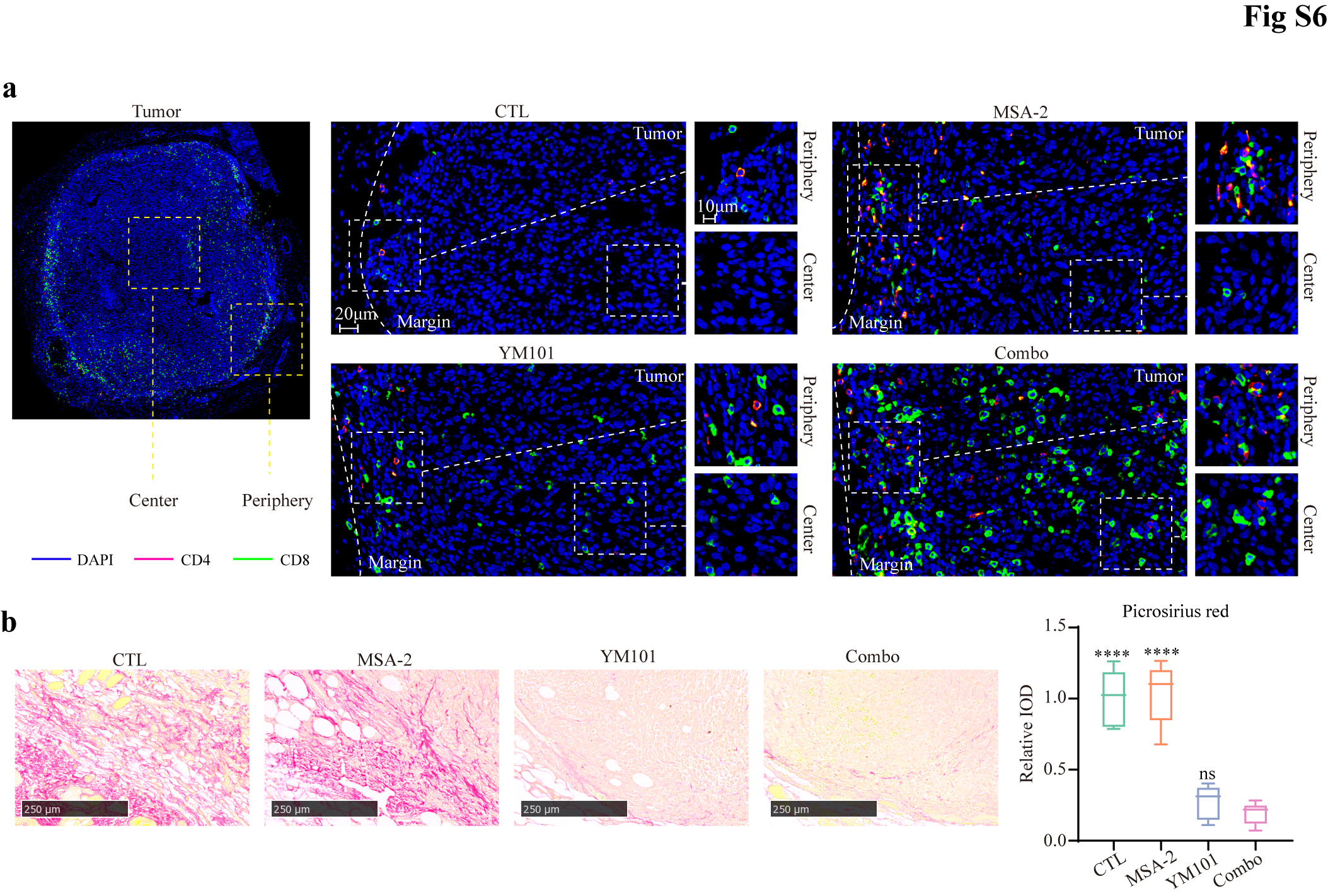

Supplement: Supplementary file 6 — Additional file 6. Figure S6: The effect of the combination therapy on T cell infiltration and peritumoral collagen deposition in the EMT-6 model. (a) Immunofluorescent staining showing T cells in tumor margin and center. (b) Picrosirius red staining showing picrosirius red staining. [file 13045_2022_1363_MOESM6_ESM.jpg]

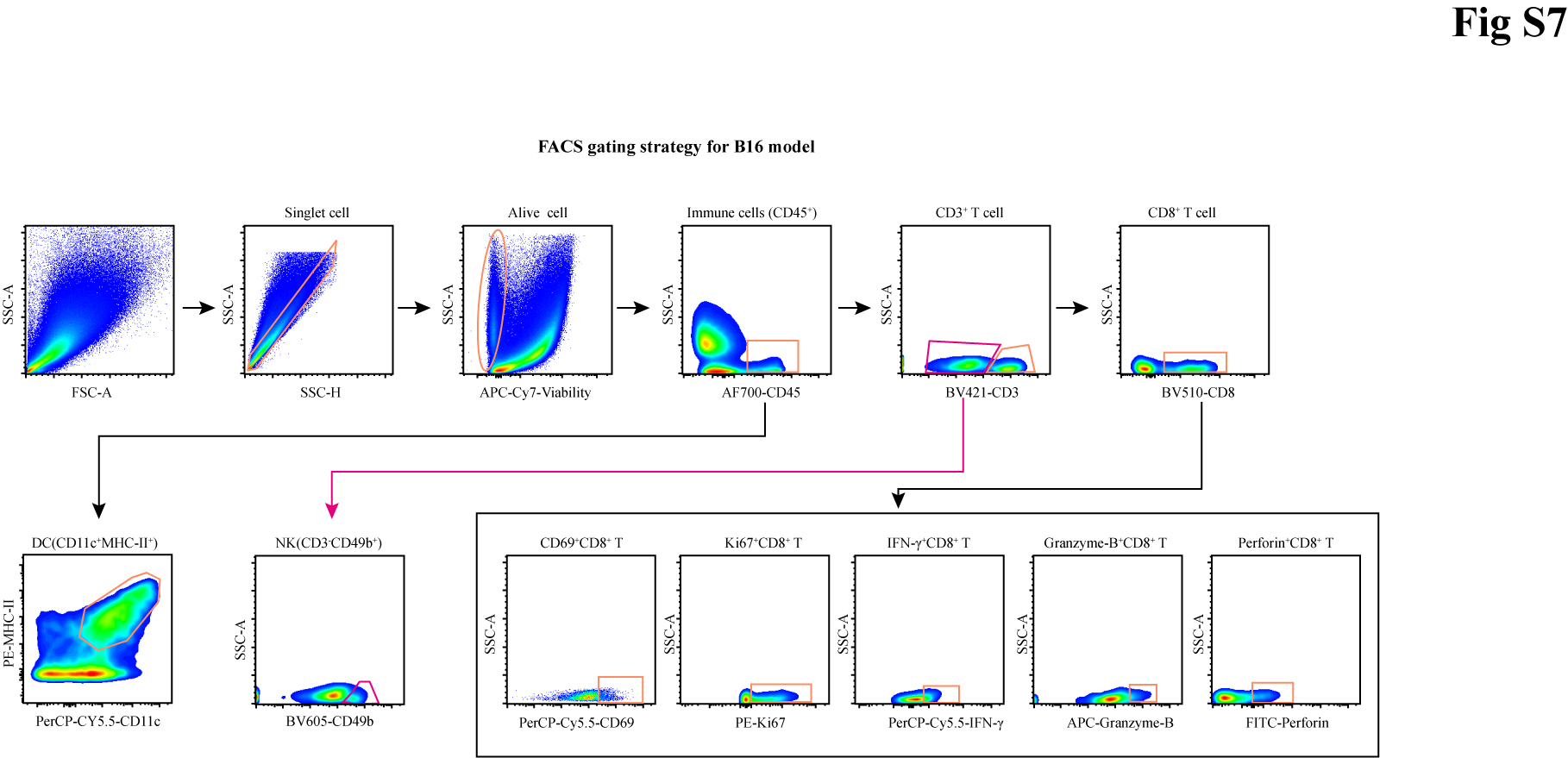

Supplement: Supplementary file 7 — Additional file 7. Figure S7: The FACS gating strategies for the B16 model. [file 13045_2022_1363_MOESM7_ESM.jpg]

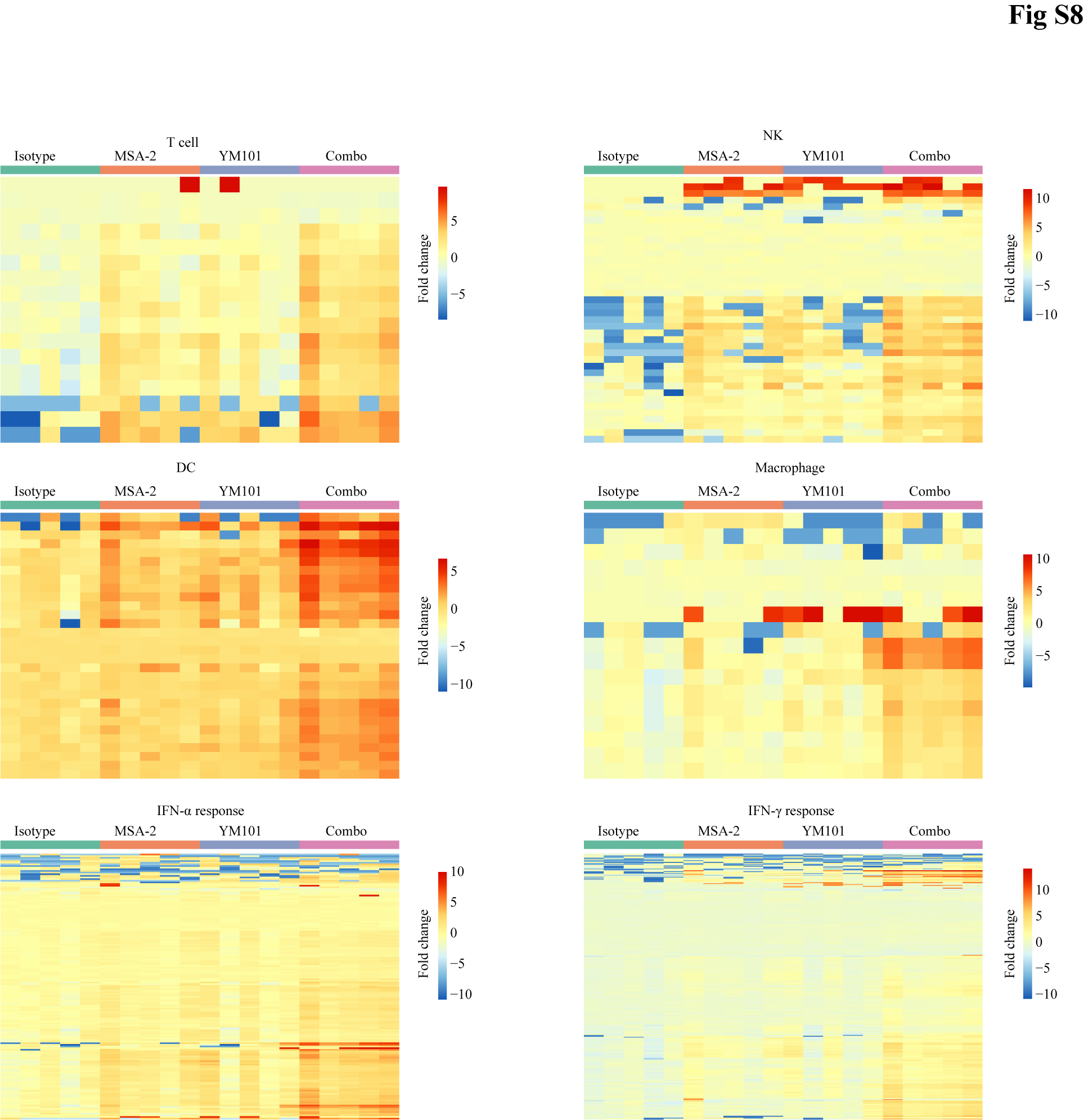

Supplement: Supplementary file 8 — Additional file 8. Figure S8: MSA-2 combined with YM101 therapy promoted immunity-associated gene expression in B16 model. The heatmaps showing the change folds of genes constituting immune signatures. [file 13045_2022_1363_MOESM8_ESM.jpg]

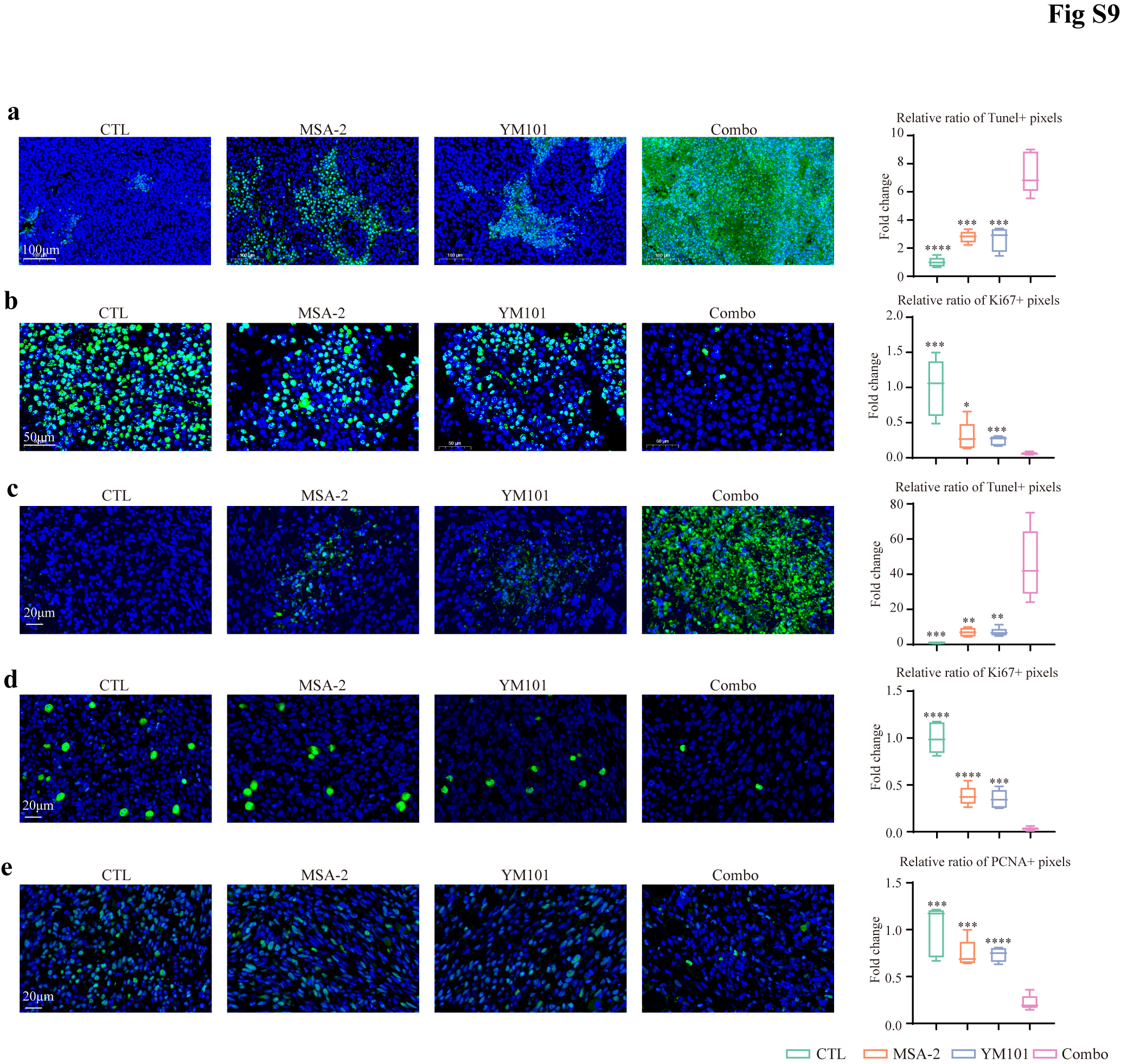

Supplement: Supplementary file 9 — Additional file 9. Figure S9: The effect of the combination therapy on proliferation and apoptosis markers in B16 and EMT-6 model. (a) Tunel staining in B16 model. (b) Ki67 staining in B16 model. (c) Tunel staining in EMT-6 model. (d) Ki67 staining in EMT-6 model. (e) PCNA staining in EMT-6 model. [file 13045_2022_1363_MOESM9_ESM.jpg]

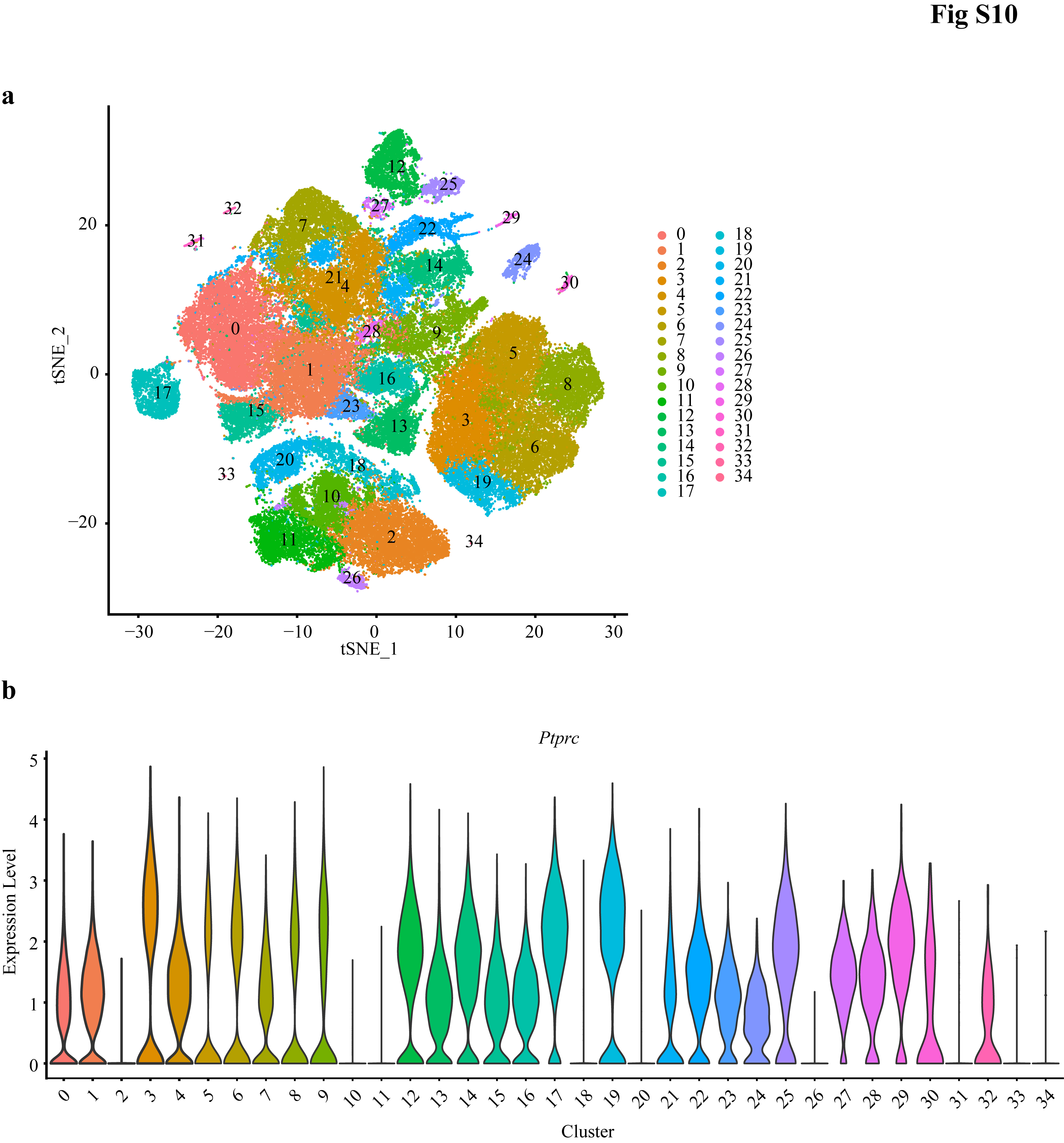

Supplement: Supplementary file 10 — Additional file 10. Figure S10. (a) T-Distributed Stochastic Neighbor Embedding (t-SNE) plot depicting clusters of all immune and non-immune cells from 24 EMT-6 tumors analyzed by 10× genomics scRNA-seq. (b) Violin plot showing expression of Ptprc (encoding CD45) in all clusters from EMT-6 tumors. [file 13045_2022_1363_MOESM10_ESM.jpg]

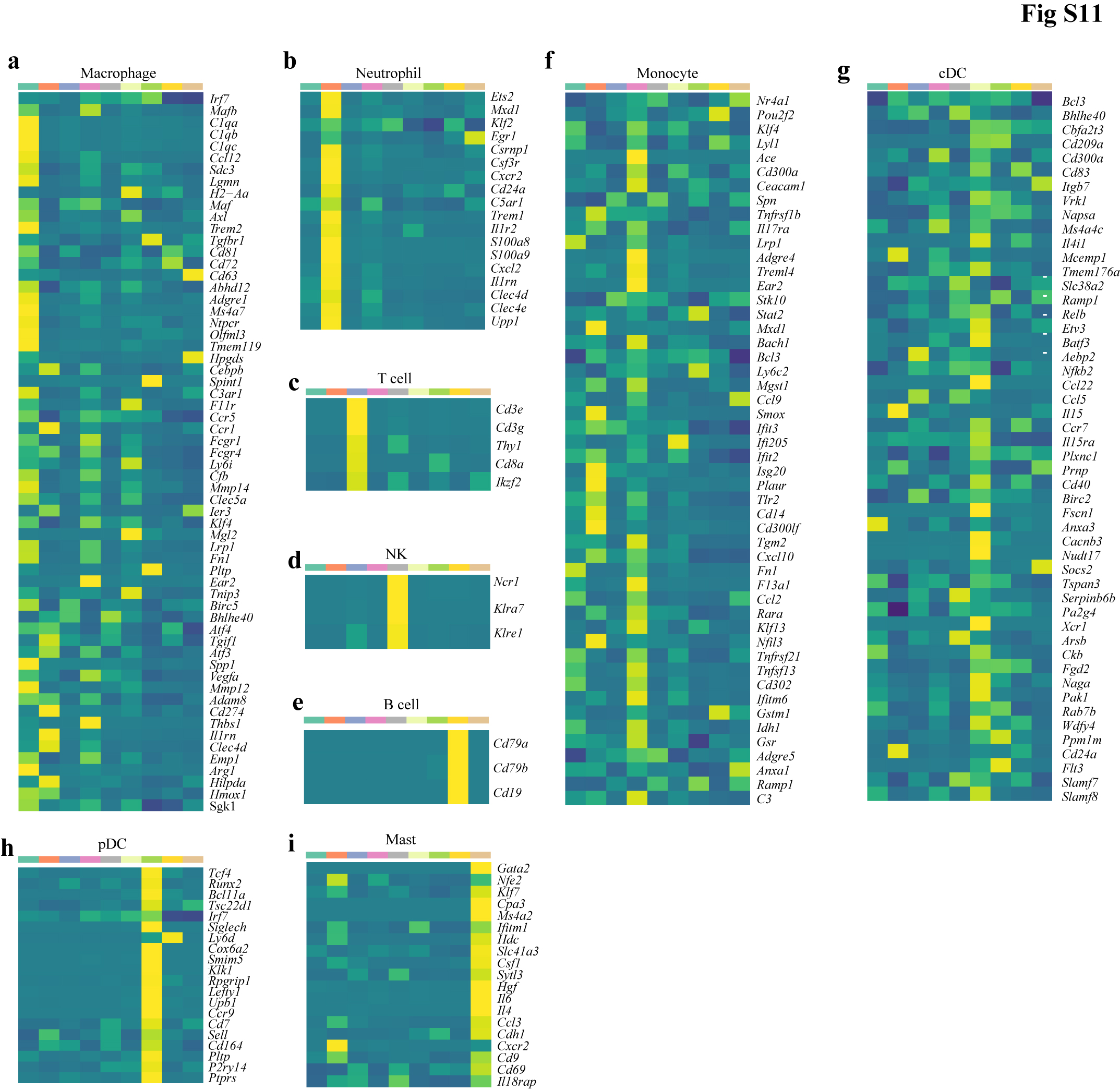

Supplement: Supplementary file 11 — Additional file 11. Figure S11. Heatmap showing cluster-specific gene profiles. (a) Macrophage-specific gene profiles. (b) Neutrophil-specific gene profiles. (c) T cell-specific gene profiles. (d) NK cell-specific gene profiles. (e) B cell-specific gene profiles. (f) Monocyte-specific gene profiles. (g) cDC-specific gene profiles. (h) pDC-specific gene profiles. (i) Mast-specific gene profiles. [file 13045_2022_1363_MOESM11_ESM.jpg]

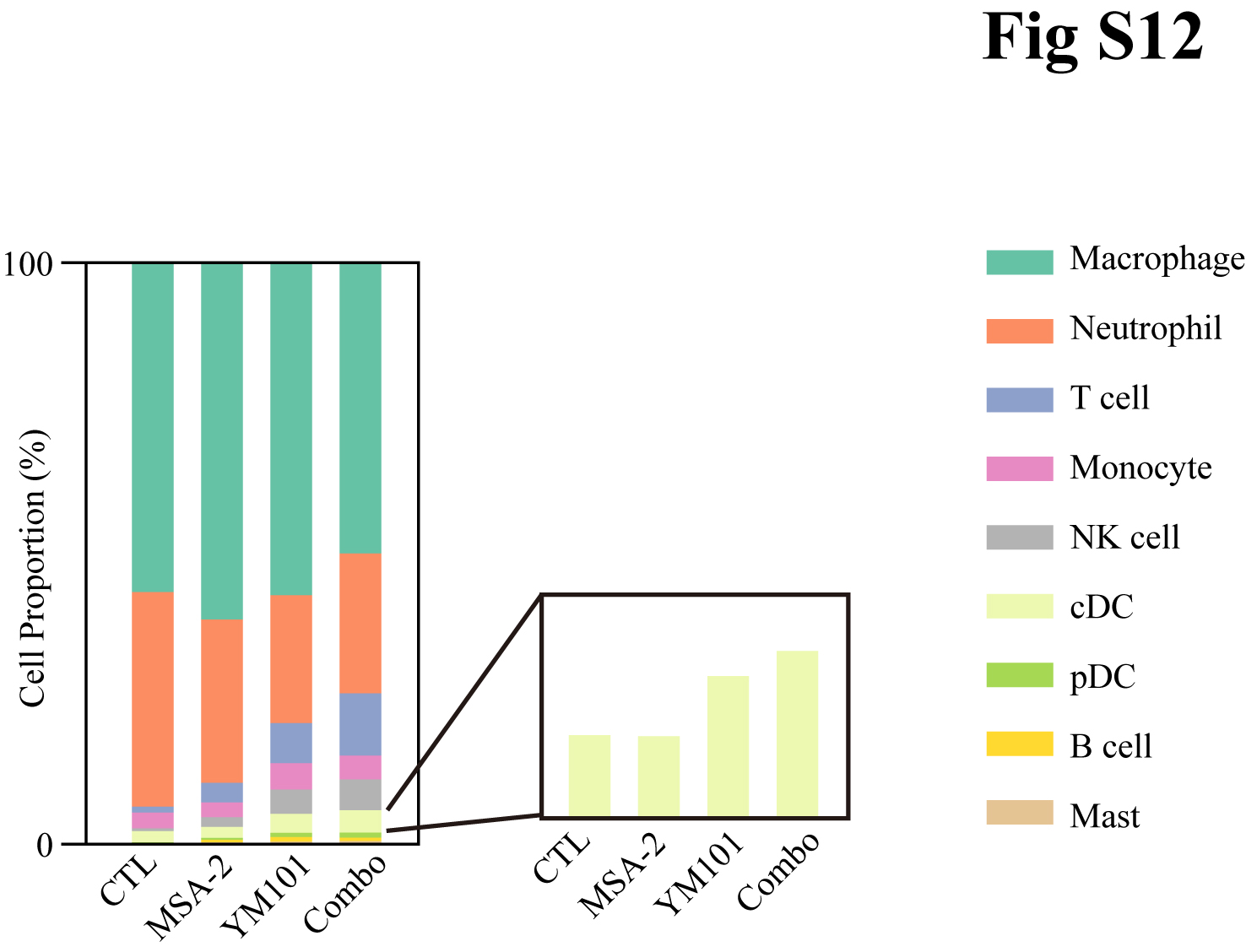

Supplement: Supplementary file 12 — Additional file 12. Figure S12. Histogram representing the proportion of clusters in each group. [file 13045_2022_1363_MOESM12_ESM.jpg]

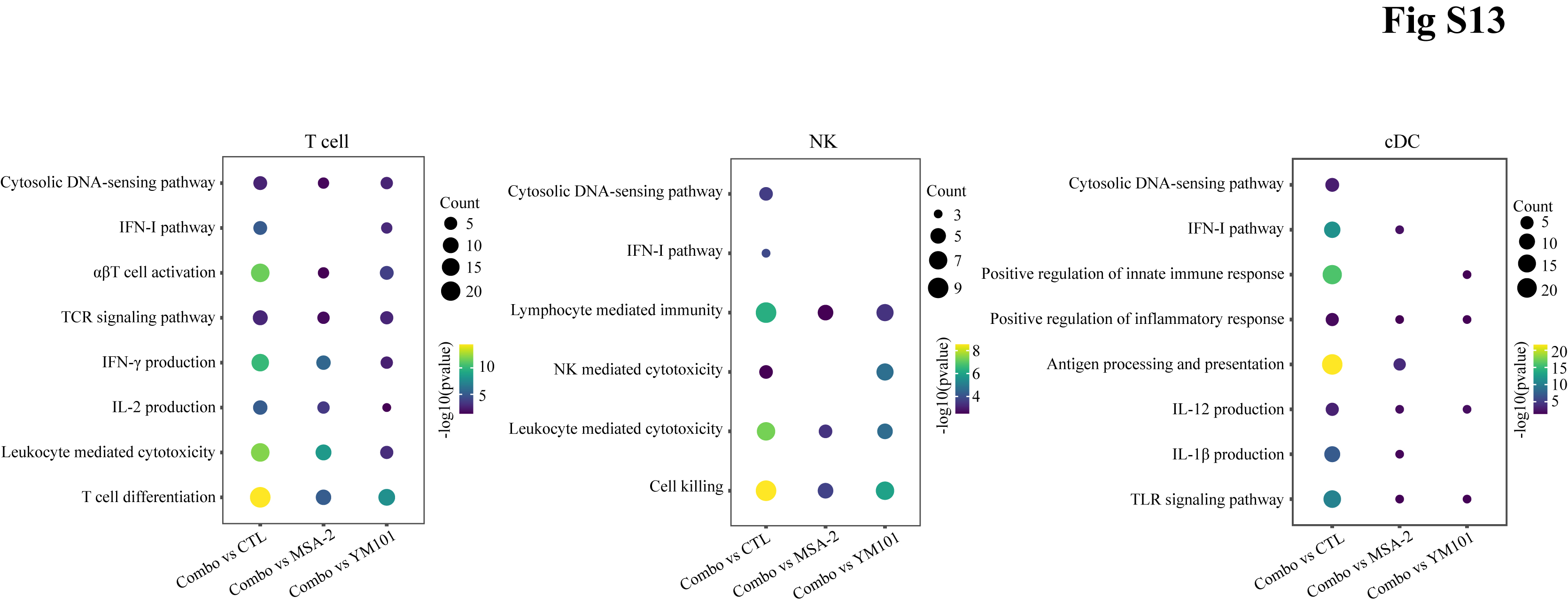

Supplement: Supplementary file 13 — Additional file 13. Figure S13. Bubble plots showing the results of DEG functional enrichment analysis using GO gene sets in T cells, NK cells, and cDCs. [file 13045_2022_1363_MOESM13_ESM.jpg]

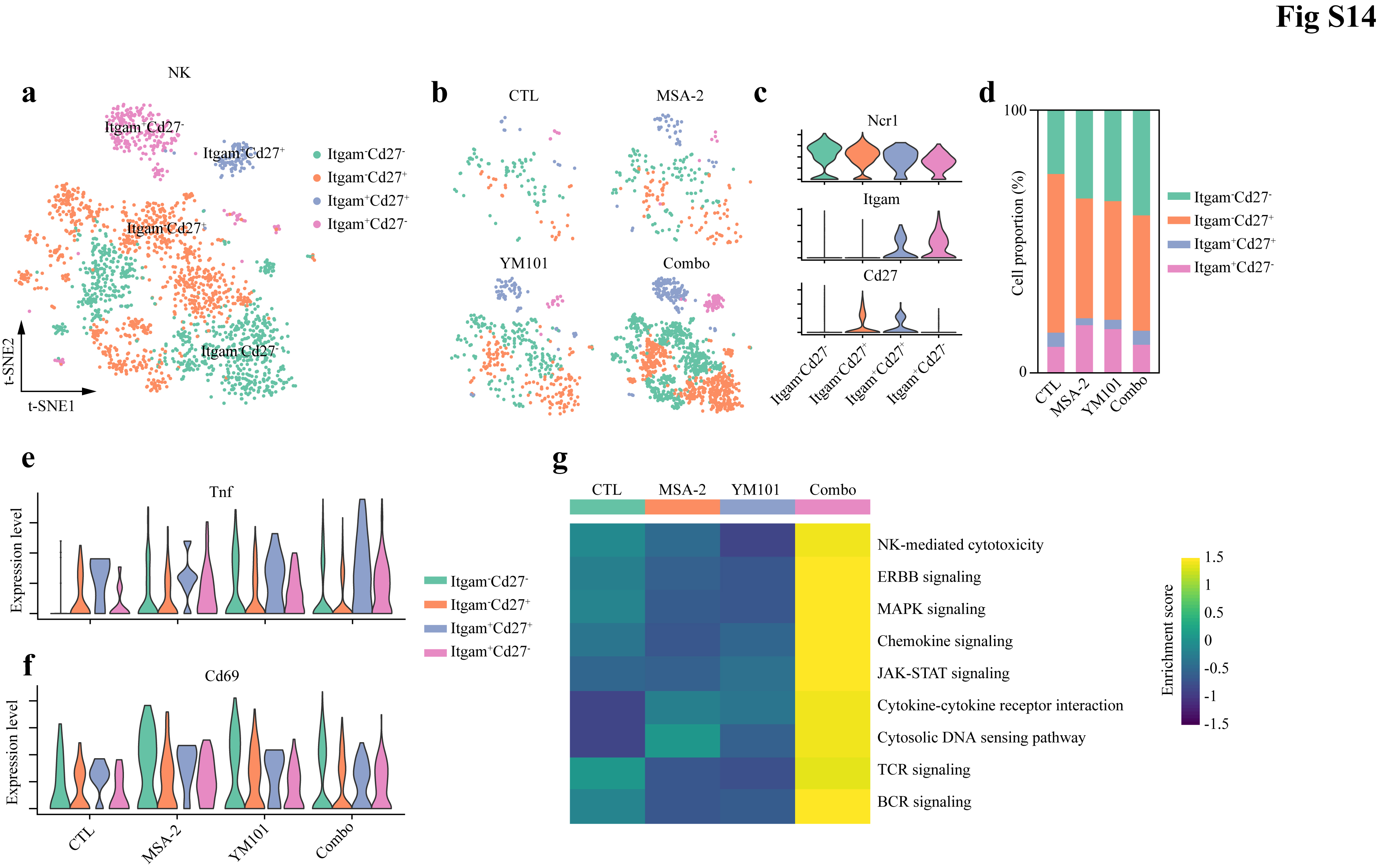

Supplement: Supplementary file 14 — Additional file 14. Figure S14. Reclustering analysis of tumor-infiltrating NK cells. (a) T-distributed stochastic neighbor embedding (t-SNE) plot for NK cells. (b) t-SNE plot for the treatment group-specific distribution of NK cell subclusters. (c) Violin plots showing NK cell subcluster-specific gene profiles. (d) Histogram representing the proportion of NK cells subcluster in each group. (e-f) Violin plots showing the levels of Cd69 and Tnf of NK cell subclusters. (g) Heatmap depicting the results of GSEA using KEGG gene sets for Itgam+ Cd27-NK cell subclusters. [file 13045_2022_1363_MOESM14_ESM.jpg]

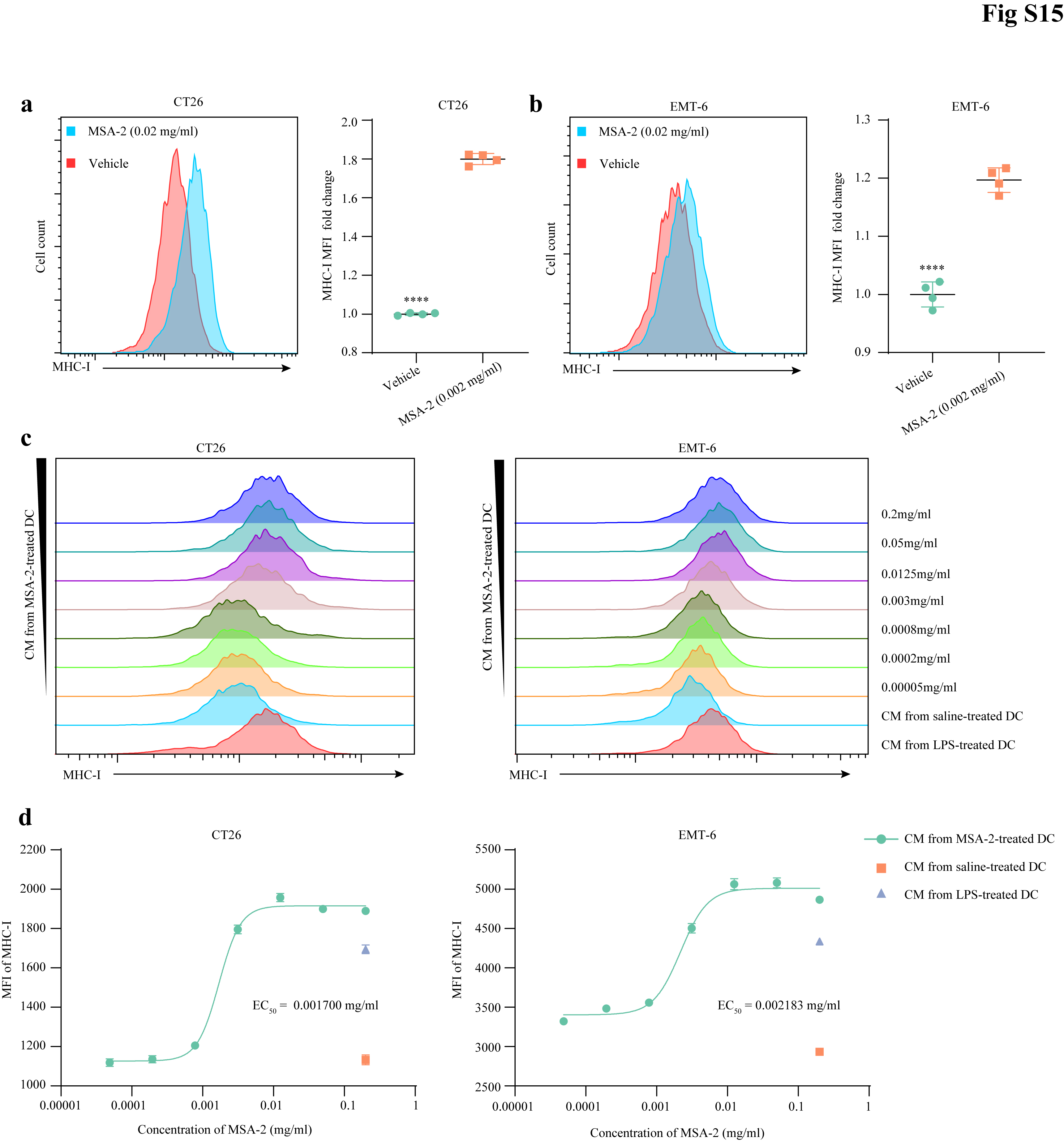

Supplement: Supplementary file 15 — Additional file 15. Figure S15. Cross talk between cancer cells and BMDCs. BMDCs were treated with 200 ng/ml LPS or different doses of MSA-2. One day later, the supernatant was discarded, and fresh medium was added. The next day, the conditioned medium from BMDCs was used for CT26 and EMT-6 culture. A day later, cancer cells were harvested for flow cytometry assay. Abs targeting H-2Kd (742436, BD) and PD-L1 (124312, BioLegend) were used in the flow cytometry assay. [file 13045_2022_1363_MOESM15_ESM.jpg]
